# A discrete serotonergic circuit regulates vulnerability to social stress

**DOI:** 10.1038/s41467-020-18010-w

**Published:** 2020-08-24

**Authors:** Wen-Jun Zou, Yun-Long Song, Min-Yi Wu, Xiang-Tian Chen, Qiang-Long You, Qian Yang, Zheng-Yi Luo, Lang Huang, Yin Kong, Jing Feng, Dong-Xiang Fang, Xiao-Wen Li, Jian-Ming Yang, Lin Mei, Tian-Ming Gao

**Affiliations:** 1 State Key Laboratory of Organ Failure Research, Key Laboratory of Mental Health of the Ministry of Education, Guangdong-Hong Kong-Macao Greater Bay Area Center for Brain Science and Brain-Inspired Intelligence, Guangdong Province Key Laboratory of Psychiatric Disorders, Collaborative Innovation Center for Brain Science, Department of Neurobiology, School of Basic Medical Sciences, Southern Medical University, Guangzhou, 510515 China; 2 Department of Neurosciences, Case Western Reserve University, Cleveland, OH 44106 USA

**Keywords:** Stress and resilience, Limbic system, Neural circuits

## Abstract

Exposure to social stress and dysregulated serotonergic neurotransmission have both been implicated in the etiology of psychiatric disorders. However, the serotonergic circuit involved in stress vulnerability is still unknown. Here, we explored whether a serotonergic input from the dorsal raphe (DR) to ventral tegmental area (VTA) influences vulnerability to social stress. We identified a distinct, anatomically and functionally defined serotonergic subpopulation in the DR that projects to the VTA (5-HT^DR→VTA^ neurons). Moreover, we found that susceptibility to social stress decreased the firing activity of 5-HT^DR→VTA^ neurons. Importantly, the bidirectional manipulation of 5-HT^DR→VTA^ neurons could modulate susceptibility to social stress. Our findings reveal that the activity of 5-HT^DR→VTA^ neurons may be an essential factor in determining individual levels of susceptibility to social stress and suggest that targeting specific serotonergic circuits may aid the development of therapies for the treatment of stress-related disorders.

## Abstract (web summary)

Serotonin is important in depression-like behavior. Here the authors show that dorsal raphe neurons that project to the ventral tegmental area are involved in regulating stress responses in mice.

## Introduction

Stress is thought to play an important role in promoting adaptation to physical or psychological threats to achieve homeostasis^1,2^. However, prolonged stress can have deleterious and long-lasting adverse effects on brain function and may contribute to the onset of psychiatric disorders, such as pathological anxiety, posttraumatic stress disorder, and major depression^2^. Interestingly, there are prominent individual variations in responses to stress: when resilient individuals are exposed to extraordinary levels of stress, they develop adaptive responses and avoid serious mental illness, while susceptible individuals adapt poorly to stress, trauma, or more chronic forms of adversity, and express inappropriate physiological and psychological responses^3^. To account for this variation, the diathesis–stress hypothesis of psychiatric disorders proposes that some individuals’ biological predispositions make them more susceptible to the effects of stressful life events^4^. Nevertheless, on which neural circuits the stress risk factors converge to yield subthreshold changes rendering individuals vulnerable to stress remains largely unknown.

The brain’s serotonergic network is a large and complex modulatory neurotransmitter system that has been demonstrated to regulate a number of behaviors, involving food intake, impulsivity, attention, and decision making^5^. The dysregulation of serotonergic neurotransmission has long been implicated in many neuropsychiatric disorders, such as anxiety and depression^6^. Indeed, there have also been reports of serotonin deficiency in subpopulations of depression patients^7,8^, and individuals carrying mutations in the serotonin transporter gene are more likely to develop depression, following exposure to stressful events^9^. In addition, the most frequently prescribed drugs on the market today for depression and anxiety disorders target the serotonergic system^10,11^. The majority of serotonergic neurons are located in the dorsal raphe nucleus (DR), comprising up to two-thirds of total neurons in this nucleus^12^. The activity of DR serotonergic neurons has been shown to play an important role in reward-associated and emotional behaviors^13–15^, and low levels of serotonin increase susceptibility to depression-like phenotypes induced by psychosocial stress^16^. Moreover, the optogenetic silencing of DR GABA neuronal activity disinhibits serotonergic neurons and prevents the acquisition of social avoidance^17^. However, insight into the direct role of DR serotonergic neurons in stress vulnerability is still scarce.

DR serotonergic neurons receive dense inputs from a broad range of limbic structures^18–20^ and transmit information to downstream targets, such as the ventral tegmental area (VTA). The VTA is an important region in motivation and reward^21^ that undergoes aberrant dopamine neuronal adaptations following chronic social defeat stress (CSDS), leading to depression-like behavior^22^. However, a clear understanding of how upstream brain regions shape VTA neurons’ responsiveness to stress is still substantially lacking.

Recent studies have shown that a large number of serotonergic inputs from the DR to VTA dopamine neurons^19^, and that the global pharmacological inhibition or lesioning of serotonergic neurons is capable of increasing the firing rate of VTA dopamine neurons^23,24^. Projections from the DR to VTA neurons strongly mediate reward^25,26^, but their implication in the stress response has yet to be addressed. Understanding whether the DR-VTA circuit mediates the effects of social stress and how this neuronal population undergoes electrophysiological adaptations in response to stress may have important implications for treatment strategies.

Here, we demonstrated that serotonergic input from the DR to the VTA plays a prominent role in regulating CSDS-induced behavior. Specifically, we reported that susceptibility to CSDS decreased the neuronal firing and the intrinsic excitability of VTA-projecting DR serotonergic neurons (5-HT^DR→VTA^ neurons). Furthermore, we found that the optogenetic inhibition of this circuit following subthreshold social defeat stress promoted stress vulnerability in stress-naive animals, and the chronic silencing of 5-HT^DR→VTA^ neurons induced stress vulnerability in mice resilient to CSDS. Interestingly, we found enhanced excitatory synaptic inputs onto 5-HT^DR→VTA^ neurons in resilient mice, and the activation of this pathway was sufficient to confer stress resilience. Taken together, we demonstrate that VTA-projecting DR serotonergic neurons may be a key factor in the regulation of stress responses.

## Results

### 5-HT^DR→VTA^ neurons represent an independent subpopulation

**Fig. 1:**
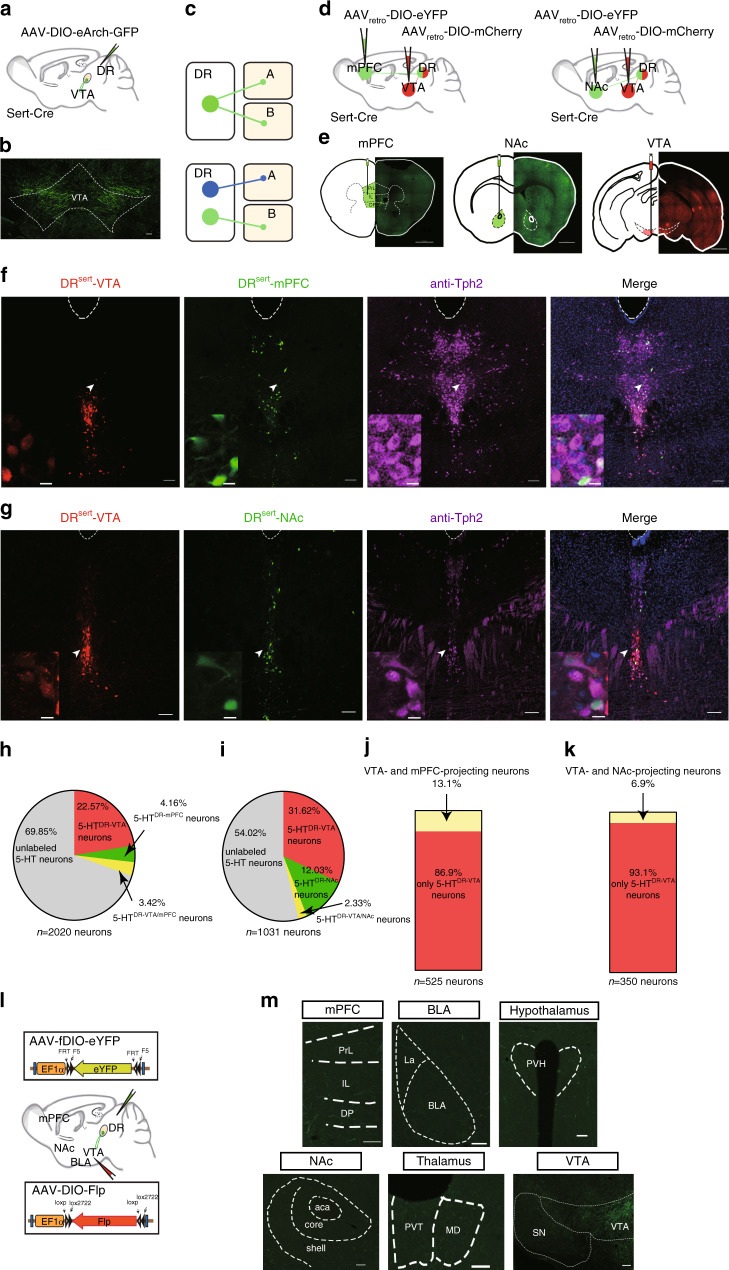
An independent subpopulation of DR 5-HT neurons project to the VTA. **a** AAV5-DIO-eArch-GFP was injected into DR of Sert-Cre mice. **b** Dense terminal expression in the VTA. Scale, 100 μm. **c** Two possible projection patterns: DR serotonergic neurons send collaterals to both A and B (top), or independent subpopulations send axons exclusively to either A or B (bottom). **d** Microinjections of AAV_retro_ carrying different fluorescent proteins (eYFP or mCherry) were placed in the mPFC and VTA (left) or the NAc and VTA (right) to retrogradely label the cell bodies of projection neurons in the DR. **e** Each section includes a schematic (left) and the histology (right) of the AAV_retro_ injection site in the mPFC (left), the NAc (center), and the VTA (right) of Sert-Cre mice. Scale, 1 mm. **f**, **g** Images of retrogradely labeled mPFC-projecting and VTA-projecting neurons (**f**), or NAc-projecting and VTA-projecting neurons (**g**) in DR. Violet, anti-Tph2 staining; green, retrogradely labeled DR-mPFC neurons (**f**) or retrogradely labeled DR-NAc neurons (**g**); and red, retrogradely labeled DR-VTA neurons. Scale, 100 μm. Insets: magnified images showing the neurons indicated with arrows in individual channels. Scale, 10 μm. **h**, **i** Pie charts depicting the percentage of mPFC-projecting only and mPFC/VTA-projecting only (**h**) (*n* = 7 sections from three mice), or NAc-projecting only and NAc/VTA-projecting only (**i**), VTA-projecting only, and AAV negative (unlabeled) in serotonergic neurons (Tph2+) in the DR (*n* = 6 sections from three mice). **j**, **k** Quantitation of dual-site retrograde tracing. The proportion of serotonergic neurons projecting to mPFC and VTA (**j**), or NAc and VTA (**k**) in 5-HT^DR→VTA^ neurons for the co-injection experiments. **l** Schematic of the viral strategy. Injection of AAV_retro_-DIO-Flp into the VTA and AAV-fDIO-eYFP into the DR of Sert-Cre mice is shown. **m** Representative coronal sections stained with GFP staining showing fibers in target areas. Scale, 100 μm. mPFC: medial prefrontal cortex, PL: prelimbic cortex, IL: infralimbic cortex, DP: dorsal peduncular cortex, NAc: nucleus accumbens, BLA: basolateral amygdala, PVT: paraventricular thalamic nucleus, MD: mediodorsal nucleus of the thalamus, PVH: paraventricular hypothalamus, SN: substantia nigra, VTA: ventral tegmental area.

To identify projection targets of DR serotonergic neurons, we labeled DR serotonergic axons by injecting a double-floxed (DIO) Cre-dependent adeno-associated virus (AAV) vector expressing enhanced archaerhodopsin (eArch) fused with green fluorescent protein (GFP; AAV-DIO-eArch-GFP) into the DR of Sert-Cre mice (Fig. 1a, Supplementary Fig. 1a, b), in which Cre recombinase was selectively expressed in adult serotonergic neurons under the control of a serotonin transporter promoter (Sert; Sert is also known as Slc6a4)^27^. DR serotonergic neurons are long-range projection neurons, as demonstrated by dense innervation in several brain areas, including the VTA (Fig. 1b, Supplementary Fig. 1c). The VTA is primarily thought to mediate stress-induced behavioral abnormalities. A key question is whether VTA-projecting DR serotonergic neurons represent an independent subpopulation (Fig. 1c). Several studies have shown that serotonergic projections from the DR to the NAc is essential for the processing of social reward^28^, and that serotonin release in the medial prefrontal cortex plays a role in driving emotional behavior^29^. To determine the identity of VTA-projecting serotonergic neurons (5-HT^DR→VTA^), we injected an axon-terminal-transducing AAV_retro_^30^ expressing Cre-dependent membrane-tethered eYFP (AAV_retro_-DIO-eYFP) or mCherry (AAV_retro_-DIO-mCherry) into the mPFC, NAc, or VTA of Sert-Cre mice (Fig. 1d, e, Supplementary Fig. 1d).

Our data demonstrated that 5-HT^DR→VTA^ neurons formed a particularly strong cluster distinct from 5-HT^DR→mPFC^ and 5-HT^DR→NAc^ clusters (Fig. 1f–i; ~22.57–31.62% 5-HT^DR→VTA^ neurons; ~4.16% 5-HT^DR→mPFC^ neurons, ~3.42% 5-HT^DR→VTA/mPFC^ neurons; ~12.03% 5-HT^DR→NAc^ neurons, ~2.33% 5-HT^DR→VTA/NAc^ neurons; ~54.02–69.85% AAV-negative serotonergic neurons). Furthermore, we found that 5-HT^DR→VTA^ neurons tended to distribute more ventrally than the other two subpopulations, with little overlap, accounting for 13.1% of the DR-mPFC volume and 6.9% of the DR-NAc volume (Fig. 1j, k). However, this methodology may not detect all axonal collateralization simultaneously because potential target regions can only be sampled one or two at a time^31^. To overcome this limitation, we used an intersectional strategy^32^ to examine the collateralization patterns of 5-HT^DR→VTA^ neurons, permitting a more comprehensive analysis of the axonal collateralization of this specific subpopulation. In Sert-Cre mice, we injected an AAV_retro_-expressing Cre-dependent Flp recombinase (AAV_retro_-DIO-Flp) into the VTA concurrently with an AAV-expressing eYFP in a Flp-dependent manner (AAV-fDIO-eYFP) into the DR (Fig. 1l, Supplementary Fig. 1e, f). In this manner, only serotonergic neurons that project to the injection site of AAV_retro_-DIO-Flp (VTA) were labeled. A detailed examination of the axonal fibers originating from 5-HT^DR→VTA^ neurons revealed that these neurons projected primarily to the VTA, with very few axonal arbors being observed in the mPFC, NAc, or others (Fig. 1m, Supplementary Fig. 1g–i).

### Stimulation of SERT fibers evokes glutamate and 5-HT effects

**Fig. 2:**
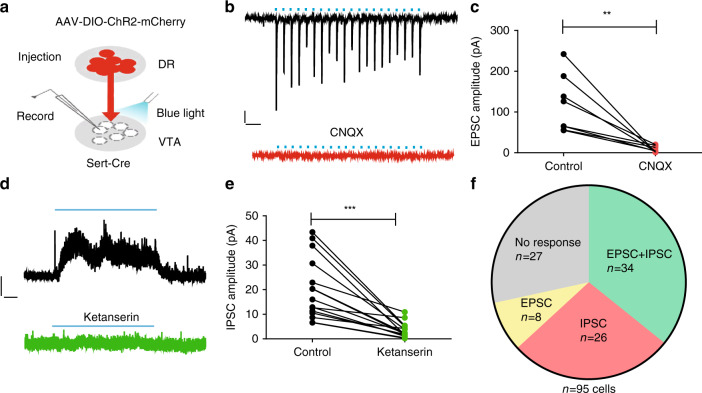
Activating serotonergic terminals elicits glutamate and 5-HT release. **a** Schematic diagram showing whole-cell patch-clamp recordings from the VTA following the Cre-dependent expression of ChR2 in the DR of Sert-Cre mice. **b** Representative recording trace from a VTA neuron showing a fast EPSC evoked by the light stimulation (5 ms, 20 Hz) of DR axonal terminals (upper panel). Stimulation-induced EPSCs were abolished by the application of CNQX, an AMPA/kainate receptor antagonist (lower panel). Scale, 50 pA,100 ms. **c** Summary data showing that the amplitudes of light-evoked EPSCs were almost completely abolished by the application of CNQX; (*n* = 8 cells from three mice; paired *t*-tests, *t*_7_ = 4.172, *P* = 0.004). **d** Example of a voltage-clamp (0 mV) trace from optogenetic stimulation (30 s 20 Hz) of ChR2+ axonal terminals produced outward current that was largely blocked by ketanserin, a 5-HT_2A_, and 5-HT_2C_ receptor antagonist. Scale, 20 pA, 4 s. **e** Summary data showing that the amplitudes of light-evoked IPSCs were significantly reduced by ketanserin (*n* = 15 cells from four mice; paired *t*-tests, *t*_14_ = 5.409, *P* = 0.000092). **f** Pie chart showing the distribution of response types in VTA neurons evoked by either single pulses (EPSC) or trains (IPSC) of optical stimulation of DR axons. EPSCs were measured at −70 mV and IPSCs at 0 mV. Data are represented as mean ± SEM; **P < 0.01, ***P < 0.001. Source data are provided as a Source Data file.

Approximately two-thirds of the serotonergic neurons in the DR co-express vesicular glutamate transporter 3 (VGluT3)^33^, a vesicular transporter that is believed to concentrate glutamate into synaptic vesicles^34^, and co-release glutamate^26,35^. To determine the neurotransmitter identity of 5-HT^DR→VTA^ neurons, we injected AAV-DIO-ChR2-mCherry into the DR of Sert-Cre animals, and examined the effect of stimulating axonal terminals from DR serotonergic neurons on postsynaptic neurons in the VTA (Fig. 2a). Single-pulse light stimulation elicited short-latency fast excitatory postsynaptic currents (EPSCs) that were completely abolished by the application of 6-cyano-7-nitroquinoxaline-2,3-dione (CNQX), a selective AMPA/kainate receptor antagonist (Fig. 2b, c). Prolonged photostimulation (20 Hz for 30 s) typically produced slow outward currents that were substantially inhibited by ketanserin (Fig. 2d, e), a drug that blocks serotonin 2 A and 2 C receptors. Interestingly, we observed that the majority of recorded VTA neurons received both AMPAR-mediated excitatory inputs and serotonin-receptor-mediated inhibitory inputs from the DR (34/95 neurons 35.8%), compared to those that exclusively received inhibitory inputs (27.4%) or exclusively received excitatory inputs (8.4%; Fig. 2f). Furthermore, tetrodotoxin (TTX; 1 μM) eliminated VTA EPSCs evoked by the light stimulation of DR inputs, which were restored by the subsequent application of 4-aminopyridine (4-AP) in the presence of TTX (Supplementary Fig. 2a–c). We also found that light-evoked EPSCs exhibited short-latency and low-response jitter (Supplementary Fig. 2d–f). From these electrophysiological findings, we inferred that VTA neurons receive direct monosynaptic inputs from DR serotonergic neurons.

### Stress induces the diminished firing of 5-HT^DR→VTA^ neurons

**Fig. 3:**
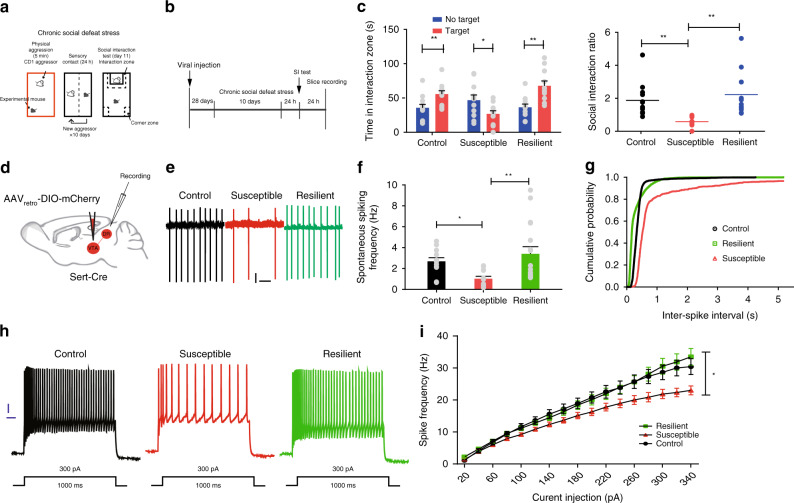
5-HT^DR→VTA^ neurons from susceptible mice show reduced firing activity. **a**, **b** Behavioral paradigm (**a**) and experimental timeline (**b**). **c** Left, time in the interaction zone in control mice (*n* = 12; one-way ANOVA*, F*_1,22_ = 8.319, *P* = 0.009) and resilient mice (*n* = 11; one-way ANOVA*, F*_1,20_ = 13.985, *P* = 0.001) was significantly increased when a target was present, whereas it was significantly decreased in susceptible mice (*n* = 11; one-way ANOVA*, F*_1,20_ = 5.125, *P* = 0.035). Right, the interaction ratio was significantly decreased in susceptible mice (control, susceptible, and resilient, *n* = 12, 11, and 11 mice; one-way ANOVA, post hoc LSD, *F*_2,31_ = 7.901, control vs susceptible: *P* = 0.005, susceptible vs resilient: *P* = 0.001). **d** Schematic for labeling 5-HT^DR→VTA^ neurons. **e** Representative firing of 5-HT^DR→VTA^ neurons from control (black), susceptible (red), and resilient (green) mice after CSDS. Scale, 20 pA, 1 s. **f** Spontaneous firing frequency of 5-HT^DR→VTA^ neurons decreased in susceptible mice compared with both control and resilient mice (*n* = 12, 12, and 17 cells from 5, 5, and 4 control, resilient, and susceptible animals, respectively; one-way ANOVA, post hoc LSD, *F*_2,38_ = 5.218, control vs susceptible: *P* = 0.046, susceptible vs resilient: *P* = 0.003). **g** Interspike intervals were calculated from each neuron firing, and the data in **f** were presented in a cumulative frequency. **h** Current-clamp traces of 5-HT^DR→VTA^ neurons from control, susceptible, and resilient animals in response to a 300 pA depolarizing step. Scale, 10 mV, 100 ms. **i** The firing frequency (Hz) of 5-HT^DR→VTA^ neurons from susceptible mice was reduced compared with both control and resilient mice, indicating reduced excitability (*n* = 14, 15, and 14 cells from 5, 4, and 4 control, resilient, and susceptible animals, respectively; two-way ANOVA, post hoc LSD, *F*_2,40_ = 3.288, control vs susceptible: *P* = 0.044, susceptible vs resilient: *P* = 0.024). Data are represented as mean ± SEM; *P < 0.05, **P < 0.01. Source data are provided as a Source Data file.

Given the multiple converging lines of evidence linking dysregulated serotonergic neurotransmission with the etiology of stress-induced affective disorders^6^, we first investigated the endogenous recruitment of the 5-HT^DR→VTA^ neurons using CSDS (Fig. 3a, b). Animals exposed to ten consecutive days of social defeat were separated into susceptible and resilient groups on the basis of their social interaction ratio score. Susceptible defeated mice showed marked social avoidance, spending significantly less time in the interaction zone, whereas resilient defeated mice displayed an interaction ratio score similar to that of undefeated control mice, spending a significant amount of time with the social target^36^ (Fig. 3c, Supplementary Fig. 3a). To label 5-HT^DR→VTA^ neurons for electrophysiological recordings, Sert-Cre animals were injected with AAV_retro_-DIO-mCherry in the VTA (Fig. 3d, Supplementary Fig. 3b–d). Next, we tested whether 5-HT^DR→VTA^ neurons exhibit aberrant electrophysiological adaptations after CSDS. Whole-cell patch-clamp recordings were performed in acute brain slices containing the DR from undefeated control, susceptible, and resilient mice. Consistent with previous reports^15,37,38^, 5-HT^DR→VTA^ neurons in control mice exhibited a firing rate of 2.68 ± 0.34 Hz (Fig. 3e, f). However, we found a decrease in the spontaneous firing rates of 5-HT^DR→VTA^ neurons in susceptible mice compared to those of control and resilient mice (Fig. 3e, f). Consistent with this finding, the interspike intervals were significantly increased (Fig. 3g). Interestingly, we also observed a significant decrease in the depolarization-evoked firing activity of 5-HT^DR→VTA^ neurons in susceptible mice compared with that in neurons of control and resilient mice (Fig. 3h, i, Supplementary Fig. 3e–g). We then measured the frequency and amplitude of spontaneous EPSCs and inhibitory postsynaptic currents (sEPSCs and sIPSCs, respectively) in these three groups. We only found an increase in the frequency of sEPSCs in 5-HT^DR→VTA^ neurons in resilient mice (Supplementary Fig. 4a, b, e). No change was observed in either the frequency of sIPSCs or in the amplitude of sEPSCs and sIPSCs (Supplementary Fig. 4c, d, f). To determine if the stress-induced alterations in synaptic inputs caused the observed excitability changes, we blocked excitatory and inhibitory synaptic currents by the application of CNQX, APV, (NMDA receptor antagonist) and bicuculline methiodide (BMI). We again found decreased excitability in susceptible mice (Supplementary Fig. 3h–k), suggesting that alterations in excitability were not due to a secondary effect of synaptic changes. Furthermore, we observed that the action potential threshold and the rheobase current were significantly increased in 5-HT^DR→VTA^ neurons of susceptible mice relative to those of control mice (Supplementary Fig. 3i, j).

### Inhibition of 5-HT^DR→VTA^ neurons promotes susceptibility

**Fig. 4:**
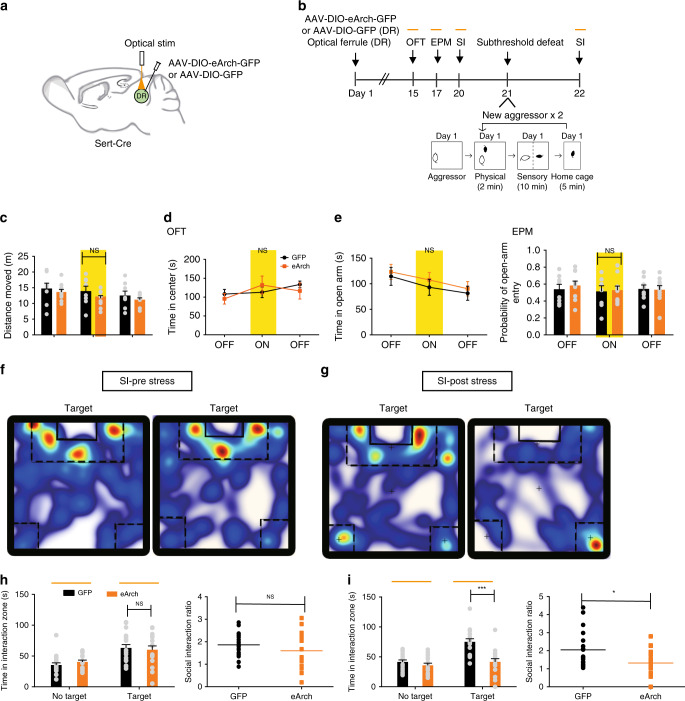
Silencing of DR 5-HT neurons induces social interaction deficits. **a** Schematic of viral injections and optic fiber implantations in the DR for optogenetic manipulation. **b** Experimental timeline of the inhibition of DR serotonergic neurons (top panel). Schematic of the subthreshold paradigm (bottom panel). **c**–**e** Silencing of DR serotonergic neurons had no effect on locomotion in the open field test (OFT; eArch, *n* = 10 mice; GFP, *n* = 8 mice; one-way ANOVA, *F*_1,16_ = 1.866, *P* = 0.191) (**c**) or anxiety-like behavior in the OFT (eArch, *n* = 10 mice; GFP, *n* = 8 mice; one-way ANOVA, *F*_1,16_ = 0.435, *P* = 0.519) (**d**), or elevated plus maze (EPM; eArch, *n* = 10 mice; GFP, *n* = 8 mice; one-way ANOVA, time: *F*_1,16_ = 0.452, *P* = 0.511, probability: *F*_1,16_ = 0.31*, P* = 0.862) (**e**). **f**, **g** Representative traces of animals expressing eArch in DR serotonergic neurons during the social interaction test with light stimulation (**f**) or after subthreshold defeat stress with optical stimulation (**g**). Warmer colors indicate more time spent. **h** No significant difference was observed for time spent in the interaction zone (eArch, *n* = 17 mice; GFP, *n* = 19 mice; one-way ANOVA, *F*_1,34_ = 0.126, *P* = 0.724) or the social interaction ratio during the silencing of DR 5-HT neurons; (eArch, *n* = 17 mice; GFP, *n* = 19 mice; one-way ANOVA, *F*_1,34_ = 1.351, *P* = 0.253). **i** Inhibition of DR serotonergic neurons following subthreshold defeat stress exposure elicits social aversion; (eArch, *n* = 17 mice; GFP, *n* = 19 mice; one-way ANOVA, interaction: *F*_1,34_ = 20.751, *P* < 0.001, SI ratio: *F*_1,34_ = 6.037, *P* = 0.019). Data are represented as mean ± SEM. NS: not statistically significant; *P < 0.05, ***P < 0.001. Source data are provided as a Source Data file.

The observation that the spontaneous firing rate and the intrinsic excitability of 5-HT^DR→VTA^ neurons were decreased in susceptible mice prompted us to hypothesize that reducing DR serotonergic neural activity may promote stress susceptibility. To test this hypothesis, we injected AAV-DIO-eArch-eYFP into the DR of Sert-Cre mice and implanted optic fibers above the DR (Fig. 4a, b, Supplementary Fig. 5a, e). The optogenetic silencing of DR serotonergic neuronal activity had no effect on locomotor activity, anxiety, or social interactions (Fig. 4c–f, h, Supplementary Fig. 5b). Interestingly, we found that mice exposed to subthreshold defeat^22^ followed by the inhibition of DR serotonergic neuronal activity showed robust social avoidance (Fig. 4g, i, Supplementary Fig. 5c). In contrast, subthreshold social stress alone did not induce social avoidance (Supplementary Fig. 5d).

**Fig. 5:**
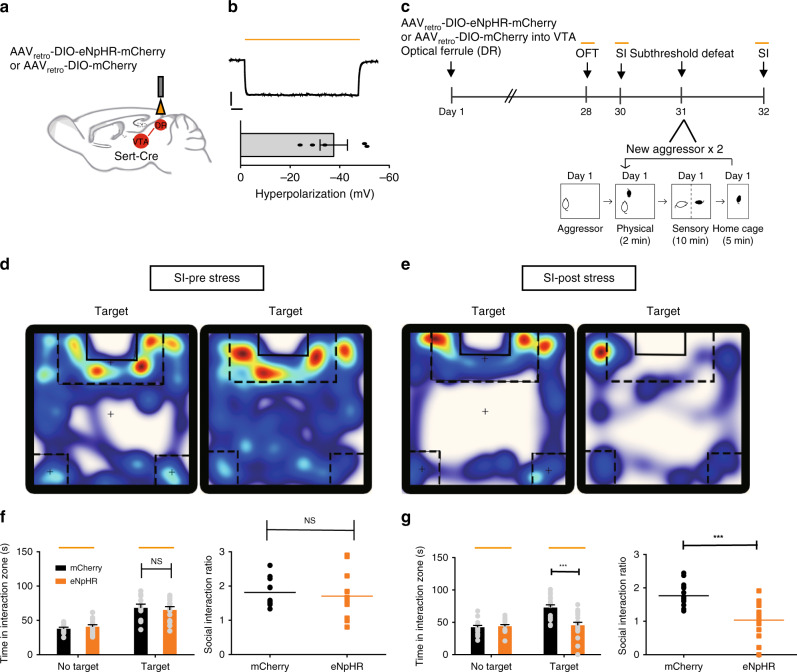
Silencing of 5-HT^DR→VTA^ neurons promotes stress susceptibility. **a** Experimental setup for the expression of AAV_retro_-DIO-eNpHR-mCherry or control virus (mCherry) in 5-HT^DR→VTA^ neurons and ferrule implantation into the DR. **b** Whole-cell current-clamp recordings of eNpHR-mCherry neurons in response to 593 nm light (yellow lines) in DR slices showed that yellow light drove hyperpolarization (top). Summary plot (bottom) showing robust light-evoked hyperpolarization (*n* = 5 neurons from two mice). Scale, 20 mV, 1 s. **c** Experimental timeline of the inhibition of 5-HT^DR→VTA^ neurons (top panel) and a schematic of the subthreshold paradigm (bottom panel). **d**, **e** Representative traces of animals expressing eNpHR in 5-HT^DR→VTA^ neurons during the social interaction test with light stimulation (**d**) or after subthreshold defeat stress with optical stimulation (**e**). Warmer colors indicate more time spent. **f** No significant difference was observed in social interaction time (eNpHR, *n* = 13 mice; mCherry, *n* = 11 mice; one-way ANOVA, *F*_1,22_ = 0.177, *P* = 0.678) or social interaction ratio during inhibition of 5-HT^DR→VTA^ neurons (eNpHR, *n* = 13 mice; mCherry, *n* = 11 mice; one-way ANOVA, *F*_1,22_ = 0.220, *P* = 0.644). **g** Silencing of 5-HT^DR-VTA^ neurons following subthreshold defeat stress exposure elicits social aversion; (eNpHR, *n* = 18 mice; mCherry, *n* = 17 mice; one-way ANOVA, interaction: *F*_1,33_ = 18.287, *P* < 0.001, SI ratio: *F*_1,33_ = 28.155, *P* < 0.001). Data are represented as mean ± SEM. NS: not statistically significant; **P < 0.01, ***P < 0.001. Source data are provided as a Source Data file.

To determine whether DR serotonergic neuronal activity promotes stress susceptibility through projections to VTA, we injected AAV_retro_-DIO-eNpHR-mCherry into the VTA of Sert-Cre mice (Fig. 5a, c). Immunohistofluorescence and electrophysiological results confirmed the viability of using AAV_retro_-DIO-eNpHR-mCherry or control vectors to express functional eNpHR in DR cells projecting to the VTA (Fig. 5b, Supplementary Fig. 3b–d). Similar to somatic manipulations, the silencing of 5-HT^DR→VTA^ neurons did not alter baseline measures of locomotion, anxiety, and social interactions (Fig. 5d, f, Supplementary Fig. 6a–c). Moreover, inhibition of 5-HT^DR→VTA^ neurons in stress-naive mice did not induce an effect in the tail suspension test (TST) or sucrose preference test (SPT; Supplementary Fig. 7a, b). However, the optogenetic inhibition of 5-HT^DR→VTA^ neurons following subthreshold exposure to aggressive CD1 mice was sufficient to induce a social avoidance phenotype similar to that seen in susceptible animals (Fig. 5e, g, Supplementary Fig. 6d). In contrast, silencing of this circuit following subthreshold social defeat stress was insufficient to induce changes in TST or SPT (Supplementary Fig. 7c, d).

### 5-HT^DR→VTA^ pathway bidirectionally regulates susceptibility

**Fig. 6:**
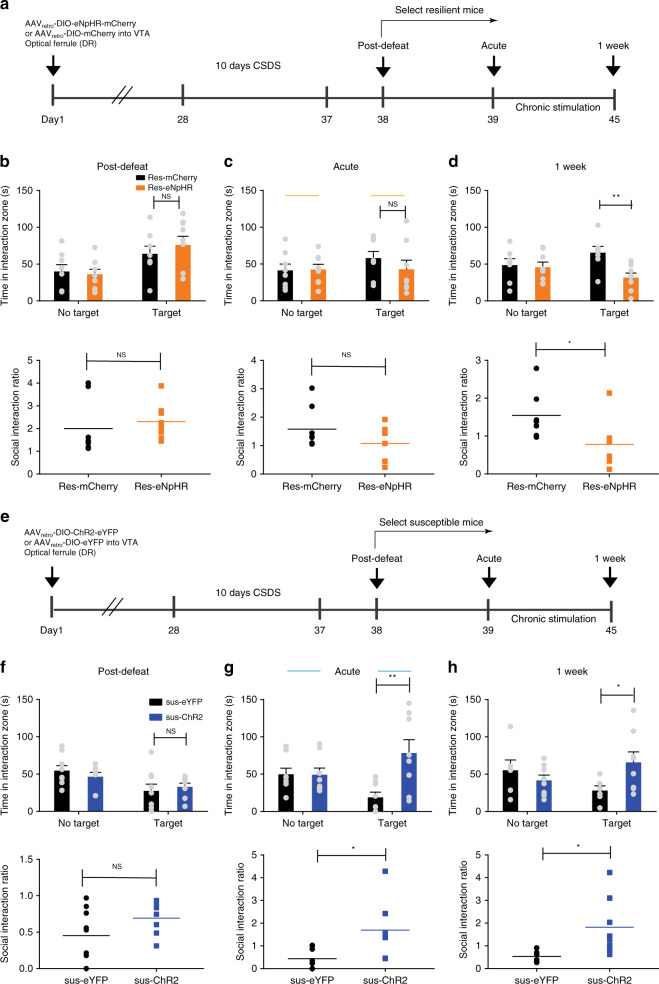
5-HT^DR→VTA^ neurons bidirectionally modulate stress susceptibility. **a**, **e** Experimental timeline and optogenetic stimulation protocol. **b**, **f** Quantitative data showing no difference in social interaction time or social interaction ratio between the resilient-eNpHR group and the mCherry group (**b**, eNpHR, *n* = 9 mice; mCherry, *n* = 8 mice; one-way ANOVA, interaction: *F*_1,15_ = 0.594, *P* = 0.453, SI ratio: *F*_1,15_ = 0.407, *P* = 0.533) or between the susceptible-channelrhodopsin-2 (sus-ChR2) group and the susceptible-enhanced yellow fluorescent protein (sus-eYFP) group (**f**, eYFP, *n* = 9 mice; ChR2, *n* = 8 mice; one-way ANOVA, interaction: *F*_1,15_ = 0.241, *P* = 0.63, SI ratio: *F*_1,15_ = 2.62, *P* = 0.126). **c**, **d** One week of the inhibition of 5-HT^DR→VTA^ neurons in resilient mice counteracted this phenotype to induce a susceptible-like phenotype (**d**, eNpHR, *n* = 8 mice; mCherry, *n* = 7 mice; one-way ANOVA, interaction: *F*_1,13_ = 10.613, *P* = 0.006, SI ratio: *F*_1,13_ = 5.546, *P* = 0.035), whereas acute stimulation during the social interaction test had no effect; (**c**, eNpHR, *n* = 8 mice; mCherry, *n* = 8 mice; one-way ANOVA, interaction: *F*_1,14_ = 0.95, *P* = 0.346, SI ratio: *F*_1,14_ = 2.231, *P* = 0.157). **g**, **h** The time spent in the interaction zone with the target of ChR2-stimulated susceptible mice was significantly increased during the acute (**g**, eYFP, *n* = 8 mice; ChR2, *n* = 8 mice; one-way ANOVA, interaction: *F*_1,14_ = 9.497, *P* = 0.008, SI ratio: *F*_1,14_ = 7.375, *P* = 0.017) or chronic stimulation of 5-HT^DR→VTA^ neurons (**h**, eYFP, *n* = 6 mice; ChR2, *n* = 8 mice; one-way ANOVA, interaction: *F*_1,12_ = 4.773, *P* = 0.049, SI ratio: *F*_1,12_ = 5.989, *P* = 0.031). Data are represented as mean ± SEM. NS: not statistically significant. *P < 0.05, **P < 0.01. Source data are provided as a Source Data file.

Given that decreased activity in 5-HT^DR→VTA^ neurons is characteristic of susceptible animals and that silencing of these neurons after subthreshold social defeat attenuates the acquisition of social interaction phenotypes, we next investigated whether the silencing of 5-HT^DR→VTA^ neurons is necessary to convert resilient animals to susceptible animals. To this end, AAV_retro_-DIO-eNpHR-mCherry or AAV_retro_-DIO-mCherry were injected into the VTA of Sert-Cre animals, and an optic ferrule was implanted above the DR (Fig. 6a, Supplementary Fig. 10b). Following a standard 10-day CSDS paradigm^39^, we identified the subgroup of mice that remained resilient based on normal social interaction ratio scores (Fig. 6b, Supplementary Fig. 8a). We found that the eNpHR-mediated inhibition of 5-HT^DR→VTA^ neurons for 5 min during a second social interaction test did not induce a significant effect on social interaction behavior (Fig. 6c, Supplementary Fig. 8b). Interestingly, chronic inhibition (20 min/day for a week) led to decreased social interactions, thereby reversing the resilient phenotype to a susceptible phenotype of defeated mice (Fig. 6d, Supplementary Fig. 8c).

To investigate whether the activation of 5-HT^DR→VTA^ neurons is sufficient to counteract vulnerability to social defeat, we injected AAV_retro_-DIO-ChR2-eYFP (yellow fluorescent protein) or AAV_retro_-DIO-eYFP into the VTA of Sert-Cre mice implanted with an optical ferrule above the DR (Fig. 6e, Supplementary Fig. 10a). We validated the specificity of viral expression in the soma and in the VTA terminals of DR serotonergic neurons (Supplementary Fig. 9a–c), and functional validation confirmed that optogenetic stimulation (20 Hz, 10 min) enables the activation of 5-HT^DR→VTA^ neurons (Supplementary Fig. 9d–g). We first identified susceptible phenotypes in a new cohort of socially defeated mice (Fig. 6f, Supplementary Fig. 8d). The optogenetic stimulation (5 ms, 20 Hz) of the 5-HT^DR→VTA^ pathway changed the behavioral phenotype of these animals from susceptible to resilient, as evidenced by a robust increase in social interactions (Fig. 6g, Supplementary Fig. 8e). This effect was also observed after 1 week of the repeated optical activation of 5-HT^DR→VTA^ neurons (twice/day, 10 min per session) triggering resilience in susceptible animals (Fig. 6h, Supplementary Fig. 8f). In control animals, neither acute nor chronic (twice/day for a week) optogenetic stimulation of DR 5-HT projections to the VTA had an effect on social interaction behavior or locomotor activity (Supplementary Fig. 11). Together, these results demonstrate that the 5-HT^DR→VTA^ circuit is not only necessary, but also sufficient for conferring vulnerability to emotional stress.

## Discussion

The dysregulation of serotonergic tone has long been implicated in the pathophysiology of stress-related neuropsychiatric disorders^6^. However, the direct contribution of the serotonergic circuit to social stress vulnerability is still unknown. Here, our results demonstrate that 5-HT^DR→VTA^ neurons mediate susceptibility to social stress and exhibit electrophysiological adaptations, a finding that supports both the serotonergic neurotransmission and the diathesis (vulnerability factors)–stress hypotheses of psychiatric disease.

Accumulating evidence suggests that the activity of serotonergic neurons is critical for the regulation of emotional states^13,14^. Findings from these studies have shown that the activation of DR serotonergic neurons elicits antidepressant-like effects and induces anxiogenic-like behaviors. However, other studies have reported that the activation of DR serotonergic neurons has no effect on anxiety-like behaviors^40,41^. While different experimental conditions and behavioral assays may contribute to these conflicting results, they may also stem from treating the DR serotonergic neurons as a single population. Here, we used viral-genetic methods to study the specific functional and anatomical properties of DR serotonergic neurons projecting to the VTA. It is well known that VTA dopamine neurons are implicated in regulating susceptibility to social stress^22^. Inputs to the VTA likely play a role in shaping stress-induced maladaptive behaviors. Indeed, a recent study has reported that parvalbumin neurons in the ventral pallidum projecting to the VTA adapt their activity in response to social stress^42^. Other studies have shown that noradrenergic inputs from the locus coeruleus to the VTA promote resilience to social stress^43,44^. The pathway from the laterodorsal tegmentum to the VTA has recently been shown to drive stress-induced maladaptive behaviors^45^. Inputs from DR serotonergic neurons to the VTA are involved in reward signaling^26,46^. The activation of this pathway has been reported to produce conditioned place preference. Our study addressed the role of VTA-projecting DR serotonergic neurons in the regulation of stress responses. Importantly, the optogenetic activation of 5-HT^DR→VTA^ neurons counteracted vulnerability to social stress. These results indicate that the DR serotonergic neuronal inputs to the VTA not only convey rewarding information, but also confer resilience to social stress. Therefore, 5-HT^DR→VTA^ neurons are a more versatile subpopulation in the DR than originally thought and should be considered a key regulator of the VTA, shaping responses to reward and stress.

Our data showed that the firing activity and intrinsic excitability of 5-HT^DR→VTA^ neurons decreased in susceptible animals, whereas the electrophysiological properties of 5-HT^DR→VTA^ neurons in resilient animals more closely resembled those of undefeated controls. This finding is consistent with a previous report indicating a significant reduction in the excitability of DR serotonergic neurons in susceptible mice^17^. Previous studies have shown that an increase in serotonin levels due to the systemic administration of the serotonin reuptake inhibitor fluoxetine inhibits the firing rate of putative dopamine neurons in the VTA^47^. In contrast, the depletion of serotonin by chemicals or electrolytic lesions of DR has been reported to increase the firing rate of VTA dopamine neurons^23,24^. Therefore, the decreased firing activity of 5-HT^DR→VTA^ neurons observed in susceptible animals may result in increasing VTA dopamine neuronal activity—a biological mark previously described in susceptible animals^22^. In line with this reasoning, the optogenetic inhibition of 5-HT^DR→VTA^ neuronal activity following subthreshold social defeat stress promoted stress vulnerability. Moreover, recent reports have shown that DR serotonergic neurons synapse on VTA dopamine neurons that heavily innervate NAc^31^^,^^46^, whereas the VTA-GABA neurons receiving input from the DR mainly target the PFC^48^. Thus, these findings are consistent with Chaudhury et al.^22^, which found that optogenetic stimulation of the VTA-NAc, but not the VTA-PFC pathway induced stress susceptibility. In contrast, Tye et al.^49^ reported that opposite effects of VTA DA neurons stimulation, reversing the susceptible phenotype to a resilient phenotype. These studies suggest that VTA DA neurons have complex and perhaps contradictory roles in the behavioral consequences of different types of stressors. It will be of interest to test whether the role of 5-HT^DR→VTA^ neurons in stress vulnerability can be extended to other experimental models. Interestingly, Wang et al.^46^ recently reported that DR dual SERT-VGluT3 terminals have an excitatory effect on some VTA dopamine neurons. Understanding the roles of specific neurons (SERT only and SERT-VGluT3 neurons) within the DR pathway to VTA might provide further insights into circuit mechanisms of stress vulnerability. We also observed that glutamate can be co-released with serotonin following the optical stimulation of 5-HT^DR→VTA^ neurons. Thus, both serotonin and glutamate may be important for controlling VTA neuronal activity. Interestingly, while decreased firing activity of 5-HT^DR→VTA^ neurons in susceptible animals was not due to alterations in synaptic inputs, we found an increase in excitatory synaptic inputs to 5-HT^DR→VTA^ neurons in resilient mice compared to levels in controls. These excitatory input adaptations in 5-HT^DR→VTA^ neurons may arise from alterations in brain areas, such as the PFC, which is often associated with processing emotional information. Previous studies have shown that cortical inputs preferentially synapse onto serotonergic neurons that are localized in the ventral DR^18–20^, where 5-HT^DR→VTA^ neurons also localize (Fig. 1). Thus, it is possible that projections from the PFC targeting 5-HT^DR→VTA^ neurons may display selective synaptic changes in resilient animals. Consistent with this notion, the optogenetic stimulation of PFC terminals in the DR or the direct activation of the DR has been shown to exert a significant antidepressant-like effect in the forced swim test^50^. Collin et al. reported an increase in the frequency of IPSCs in DR serotonergic neurons as a whole in susceptible mice^17^, while we found no change in IPSCs in 5-HT^DR→VTA^ neurons. These findings suggest that the heterogeneous physiological responses seen in DR serotonergic neurons could result from recording at least two distinct subpopulations. Future research will be required to identify the area-specific synaptic inputs onto 5-HT^DR→VTA^ neurons to understand which precise upstream pathways drive CSDS-induced alterations in resilient conditions.

The biological factors that determine whether an individual develops mental illness, such as posttraumatic stress disorder or depression, remain largely unknown. Our data demonstrate that the activation of 5-HT^DR→VTA^ neurons counteracted the reduction in social interaction time in susceptible mice, which indicates that 5-HT^DR→VTA^ neuron excitation is sufficient to confer stress resilience. Conversely, the silencing of 5-HT^DR→VTA^ neurons resulted in reduced social interaction time, which suggests that 5-HT^DR→VTA^ neuron inhibition is sufficient to induce stress susceptibility. These results highlight that inputs from DR serotonergic neurons to the VTA play a prominent role in regulating vulnerability to social stress. Consistent with this conclusion, several studies have shown that individuals carrying mutations within the serotonin transporter gene have an increased risk of major depression following exposure to stress^9^. In addition, a previous study reported that serotonin deficiency increases susceptibility to psychosocial stress^16^. In another recent study, social isolation was shown to decrease the excitability of DR serotonergic neurons^51^. Interestingly, Zhou et al.^52^ recently reported that activation of the 5-HT^DR^→SOM^CeA^ circuit reduces depressive-like behavior in mice exposed to chronic pain, but not in mice exposed to chronic stress. The finding, together with our data, suggests that different etiologies of psychiatric disorders, for example, chronic pain and stress, may be encoded by distinct serotonergic pathways. Notably, we surprisingly found that the chronic inhibition of 5-HT^DR→VTA^ neural activity in resilient mice counteracted this phenotype to induce a susceptible-like phenotype, whereas this effect was not observed after acute inhibition during the social interaction test. One possible explanation for this result is that the synaptic connection between 5-HT^DR→VTA^ neurons and VTA neurons is weak in healthy mice, but may be selectively stronger in resilient mice. It is possible that chronic inhibition, which results in long-term plasticity adaptations, may convert resilient mice into susceptible mice. In line with this reasoning, we demonstrated that 5-HT^DR→VTA^ neurons in resilient mice received increased excitatory synaptic input compared to those in susceptible and control mice.

Future interrogation into the downstream circuits and VTA cell types engaged by DR serotonergic neurons, and their neural adaptations in response to CSDS will be required.

Taken together, we characterize a neural circuit that mediates vulnerability to chronic social stress, a finding that could have important implications for our understanding of the etiology of psychosocial stress, and suggests that future strategies aimed at activating the 5-HT^DR→VTA^ neurons may be protective against stress-related psychiatric disorders.

## Methods

### Mice

All animal procedures were performed in accordance with institutional guidelines of Southern Medical University, Guangzhou, and the governmental regulations of China. We used (all males, aged 8–12 weeks) Sert-Cre mice (strain name B6.Cg-Tg(Slc6a4-Cre)ET33Gsat; a gift from Minmin Luo at NIBS). Male C57BL/6 J mice (aged 8–10 weeks) were obtained from the Southern Medical University Animal Center (Guangzhou, China). All mice were maintained on a 12-h light–dark cycle with ad libitum access to food and water.

### Virus

AAVs used in this study were purchased from BrainVTA, Wuhan, China and included AAV_retro_-Ef1α-DIO-eNpHR3.0-mCherry (titer, 5.00 × 10^12^ v.g./mL), AAV_retro_-Ef1α-DIO-mCherry (titer, 5.63 × 10^12^ v.g./mL), AAV_retro_-nEf1α-FDIO-eYFP (titer, 5.28 × 10^12^ v.g./mL), and AAV_retro_-Ef1α-DIO-FLP (titer, 5.38 × 10^12^ v.g./mL). AAV5-Ef1α-DIO-ChR2-mCherry (titer, 3.64 × 10^12^ v.g./mL), AAV5-Ef1α-DIO-Cherry (titer, 1.42E × 10^12^ v.g./mL), AAV5-Ef1α-DIO-eArch-GFP (titer, 5.54 × 10^12^ v.g./mL), and AAV5-Ef1α-DIO-GFP (titer, 3.81 × 10^12^ v.g./mL) were purchased from Sunbio Medical Biotechnology, Shanghai, China. AAV_retro_-Ef1α-DIO-hChR2-eYFP (titer, 1.56 × 10^13^ v.g./mL) and AAV_retro_-Ef1α-DIO -eYFP (titer, 1.59 × 10^13^ v.g./mL) were purchased from Taitool Bioscience, Shanghai, China.

### Viral injections and stereotaxic surgeries

For all injections, mice were anesthetized using pentobarbital (intraperitoneal, 75 mg/kg) and placed in a small animal stereotaxic instrument equipped with a heating pad. Hair was removed from the dorsal surface of the head with hair clippers, and ophthalmic ointment was applied to the eyes. Craniotomies were performed above the target region, and all measurements were made relative to bregma for virus/implant surgeries. Injections were performed using a microsyringe pump (Nanoliter 2010 Injector, WPI) at a rate of 100 nl/min. The glass pipette was retracted after 5–10 min and then slowly withdrawn.

To label DR 5-HT axons, we injected 500 nL of AAV5-ef1α-DIO-GFP into the DR of Sert-Cre mice at the following coordinates: anteroposterior (AP): 5.2 mm, mediolateral (ML): 0 mm, and dorsoventral (DV): 2.6 mm (a 15° angle from caudal to rostral); for retrograde tracing, we bilaterally injected 300–500 nL of the following viruses: AAV_retro_ -Ef1α-DIO-mCherry and AAV_retro_-Ef1α-DIO-eYFP into the VTA (AP: −3.1 mm, ML: ±0.5 mm, and DV: −4.6 mm), NAc (AP: +1.6 mm, ML: ±0.7 mm, and DV: −4.2 mm), and/or mPFC (AP: +1.8 mm, ML: ±0.3 mm, and DV: −2.3 mm) of Sert-Cre mice.

To express opsins in the DR serotonergic neurons inhibition experiments, 500 nL of AAV5-Ef1α-DIO-eArch-GFP or AAV5-Ef1α-DIO-GFP was infused into the DR of Sert-Cre mice. To specifically target DR neurons projecting to the VTA, Sert-Cre mice aged ~8 weeks were injected with 500 nL of AAV_retro_-Ef1α-DIO-eNpHR3.0-mCherry, AAV_retro_-Ef1α-DIO-mCherry, AAV_retro_ -Ef1α-DIO-ChR2-eYFP, or AAV_retro_-Ef1α-DIO -eYFP into the VTA. Ferrules attached to optical fibers (200 mm core diameter, 0.37 numerical aperture (NA)) were implanted 0.2 mm above the DR for somatic stimulation. Four weeks were given for the full expression of viruses in somas before behavioral testing commenced. Upon the completion of the behavioral experiments, coronal sections of DR were used to confirm viral expression and fiber placement.

Approximately, 8-week-old Sert-Cre mice were injected with AAV5-Ef1α-DIO-ChR2-mCherry into the DR using the same volumes and coordinates as previously stated. A total of 6–8 weeks were to ensure for viral expression in terminals before animals were sacrificed for electrophysiology. For the retrograde labeling of VTA neurons projecting to DR, AAV_retro_-Ef1α-DIO-mCherry was infused into the VTA of Sert-Cre mice. Electrophysiological recordings were performed 5–6 weeks after stereotaxic surgery, allowing the full expression of viruses.

### Histology and imaging

Mice were anesthetized with pentobarbital (intraperitonial (i.p.) injection) and then intracardially perfused with 0.9% saline, followed by fixation with 4% paraformaldehyde (PFA) in PBS. Brains were carefully removed and postfixed in 4% PFA overnight before being transferred into 30% sucrose in PBS solution until they sank to the bottom of the container. Forty-micron-thick coronal sections containing the brain region of interest were sliced on a freezing microtome (Leica CM1950).

Sections were washed with PBS three times (5 min each) and were then blocked in 5% normal goat serum solution containing 0.3% Triton X-100 for 2 h at room temperature. Then, slices were incubated with a rabbit polyclonal antibody against Tph2 (1:400, Millipore) or anti-GFP antibody (1:500, Invitrogen) in 5% bovine serum albumin at 4 °C overnight, and washed three times (5 min each) in PBS before incubation for 2 h at room temperature with Alexa Fluor-conjugated secondary antibodies (1:500, Invitrogen, goat anti-rabbit IgG (H + L) highly cross-adsorbed secondary antibody, Alexa Fluor 647 Cat# A-21245; goat anti-rabbit IgG (H + L) highly cross-adsorbed secondary antibody, Alexa Fluor 488 Cat# A-11034; and goat anti-rabbit IgG (H + L) highly cross-adsorbed secondary antibody, Alexa Fluor 594 Cat# A-11037).

We first verified that the AAV injections were localized to the target brain regions (DR, VTA, NAc, and mPFC). Brain regions were determined by anatomical landmarks, and were based on the Mouse Brain Atlas in Stereotaxic Coordinates, Paxinos and Franklin, second edition. For the quantification of starter cells, the number of Tph2^+^ and AAV-positive cells in the DR were counted in 1–2 sections out of every six, from bregma −4.24 to −4.96 mm. The cellular resolution images of coronal sections of interest were acquired through the 10× and 20× objective, using the Nikon A1 confocal microscope and were processed with NIH ImageJ software. Sixteen/22 mice were excluded from the double-site retrograde tracing experiments because of failed injection or because they did not show any dual-color AAV expression.

Images were acquired using a Nikon A1 confocal microscope with a 20× objective (NA 0.8). All images were taken in roughly the same imaging area. Images were processed using NIH ImageJ software and were quantified as a percentage of the area of thresholded pixels normalized to the AAV_retro_-DIO-Flp injection site. Qualitatively determined threshold values were maintained consistent throughout animals. Sample size was based on reports in related literature and was not predetermined by calculation.

### In vitro electrophysiological recording

The methods of slice preparation and physiological recordings were similar to those in a previous study^53^. Briefly, 10- to 12-week-old mice were deeply anesthetized with an i.p. injection of pentobarbital (100 mg/kg). Mice were then rapidly decapitated, and brains were removed from the cranial cavity and dissected into ice-cold oxygenated artificial cerebrospinal fluid (ACSF), containing (in mM) 250 sucrose, 26 NaHCO_3_, 10 glucose, 10 MgSO_4_, 2 KCl, 1.3 NaH_2_PO_4_, and 0.2 CaCl_2_ (saturated with 95% O_2_ and 5% CO_2_). Coronal brain sections (300 μm thickness) containing DR or VTA were cut using a vibratome (VT-1200S, Leica, Germany). Brain slices were incubated at 34 °C for 30 min and then at room temperature (25 ± 1 °C) to recover in a holding chamber containing oxygenated ACSF (in mM; 126 NaCl, 26 NaHCO_3_, 10 glucose, 3 KCl, 2 CaCl_2_, 1.25 NaH_2_PO_4_, and 1 MgSO_4_) before being transferred to the recording chamber for electrophysiological recordings. Neurons were visualized under an upright microscope (ECLIPSE FN1, Nikon) equipped with a 40× water-immersion lens, infrared differential interference contrast and digital camera (C11440-42U, Hamamatsu).

Electrophysiological recordings were made using a MultiClamp700B amplifier and PClamp software (Molecular Devices, USA). The data were low-pass filtered at 2 kHz and digitized at 10 kHz with Digidata 1440 (Molecular Devices, USA). During recording, slices were submerged in normal, oxygenated ACSF and superfused (2 ml/min) at temperature (32–34 °C). The pipette (4–6 MΩ) was pulled by a micropipette puller (P-97, Sutter instrument) and filled with the internal solution (in mM: 105 K-gluconate, 30 KCl, 10 HEPES, 10 phosphocreatine, 4 ATP-Mg, 0.3 GTP-Na, and 0.3 EGTA, pH 7.35, 285 mOsm). Cell-attached voltage clamp was used to record spontaneous firing of serotonergic neurons in DR slices. Unlike whole-cell patch clamping, which disturbs the intracellular contents and can thereby change the membrane potential of serotonergic neurons during recordings, cell-attached patch clamping was chosen to produce reliable, long-lasting, and stable recording of firing of serotonergic neurons^54^. Following a 3-min stabilization period, cell-attached voltage clamp was performed.

To measure the intrinsic membrane properties of DR serotonergic neurons, whole-cell recordings were carried out in current-clamp mode, and spikes were induced by incremental increases in the current injection (from −40 to 340 pA at a step of 20 pA). All action potential properties and excitability recordings in Supplementary Fig. 3h–k were performed in the presence of 20 μM CNQX, 100 μM dl-AP5, and 20 μM BMI. The action potential threshold was measured as the point of depolarization at which the neuron fires. Rheobase was determined by injecting a ramp of current and examining the current needed to elicit the first spike. Membrane resistance was calculated from the change in voltage elicited after a series of hyperpolarizing pulses.

For target recordings in the VTA, AAV-DIO-ChR2-mCherry was injected into the DR of Sert-Cre animals and cells in the VTA near mCherry + fibers were patched. For the photostimulation of ChR2-expressing axon terminals, a 5 ms blue light pulse was emitted from a Lambda DG-4 (Sutter, USA) under the control of Digidata 1440. A Lambda DG-4 Wavelength Switcher was used to deliver different wavelengths of light through the 40× objective. Recordings were conducted in voltage-clamp mode using a cesium-methansulfonate (Cs-Meth)-based internal solution (in mM: 135 Cs-Meth, 10 KCl, 1 MgCl_2_, 0.2 EGTA, 2 QX-314, 4 ATP-Mg, 0.3 GTP-Na, and 20 phosphocreatine, pH 7.3, 290–300 mOsm) so we could detect EPSCs (−70 mV) and IPSCs (0 mV) in the same neuron^42^. sEPSCs and sIPSCs were also recorded at −70 mV and 0 mV, respectively. For drug application, AMPA-mediated EPSCs were blocked by the bath application of CNQX (10 µM, Sigma), and 5-HT-receptor-mediated IPSCs were blocked with ketanserin (10 µM, Sigma). sEPSCs were validated by the addition of kynurenic acid (3 mM, Sigma), and sIPSCs were validated with bicuculine (20 µM, Sigma). For eNpHR experiments, different durations of yellow light were generated by the above mentioned stimulator and delivered to DR serotonergic neurons expressing eNpHR.

To verify the monosynaptic connection between DR 5-HT axons and VTA neurons, TTX (1 µM) and 4-AP (200 mM) were bath applied in succession. The latency of optical-evoked EPSCs was calculated by the time interval between the onset of the light pulse and the time point at which 10% of the peak amplitude was achieved. The response jitter was calculated by measuring the s.d. of the latency of five successive recordings for each VTA neuron.

In all experiments, the series resistance was controlled <30 MΩ. All recordings were excluded if the holding current exceeded −200 pA or if series resistance fluctuated >20% from the initial values. The data were acquired by pClAMP 10.7, and analyzed using Clampfit 10.7 software and Mini Analysis software.

### Behavioral assays

For ChR2-mediated stimulation experiments, optical fibers (200-μm diameter, NA 0.37, Inper, Hangzhou, China) were connected to patch cords (Thorlabs), which were, in turn, connected to blue or yellow light lasers (Thinker Tech Nanjing Biotech Co., Ltd., China) using FC/PC adapters located above the operant chambers. Laser output was controlled with a stimulator (Thinker Tech Nanjing Biotech Co., Ltd., China). For DR stimulation, 5 ms pulses of 20 Hz light were delivered as outlined in previous studies^28^ that optogenetically manipulated DR serotonergic neurons. Laser power was measured (Thorlabs) before each experiment and was measured to be ~15 mW. For NpHR-mediated inhibition, fibers were connected to a 593 nm yellow laser diode. Laser power was again measured (Thorlabs) before each experiment and adjusted to ~8 mW.

### Chronic social defeat stress

CSDS protocal was conducted according to the model described by Golden et al.^55^. Before the start of the social defeat experiments, retired male breeder CD1 mice were screened on three consecutive days and were selected according to the following criteria: (i) a latency of attack under 60 s, (ii) consistent bouts over 3 min long, and (iii) attack in at least the two past screening days. Experimental mice were subjected to physical contact with a novel CD1 aggressive mouse for ten consecutive days. Following daily physical defeat, the mice remained in the aggressor’s home cage on the other side of a translucent divider that was perforated with holes to allow for visual, auditory, and olfactory interaction with the aggressor for 24 h until the next physical defeat. The control mice were housed two animals per cage in the same conditions as their experimental counterparts, but were never exposed to the CD1 aggressors. Each day, the experimental and the control mice were relocated to a cage with a new cage mate immediately before social defeat. After 10 days of CSDS, all mice were housed singly and tested 24 h later for the social interaction test.

### Subthreshold social defeat stress

The subthreshold social defeat experiment is a well-established protocol^22^. Briefly, an experimental mouse was introduced into a CD1 aggressor’s home cage for two consecutive bouts of 3 min of physical defeat on a single day. After 3 min of physical defeat, the mouse was kept with the CD1 aggressor separated by a perforated partition for 10 min. A 5 min rest in the home cage was given between the sensory defeat session and the subsequent bout of physical defeat in the home cage of a new CD1 aggressor. Social defeat was repeated once, and experimental mice were assessed using the social interaction test the following day.

### Social interaction test

Social interaction was measured in a two-stage social interaction test. In the first stage (target absent), the experimental mice were placed in an open field arena (42 cm × 42 cm × 42 cm) with an empty wire mesh enclosure (10 cm wide × 6.5 cm deep × 42 cm high) in the middle of one of the arena walls. In the second stage, the experimental mouse was reintroduced into the arena, and a novel, aggressive CD1 mouse was placed into the wire mesh enclosure. Between the two stages, animals were returned to home cage for 30 s. In all behavioral experiments, animals were monitored with video tracking software (Ethovision XT 10, Noldus). Time spent in the social interaction zone, which was defined to as a 24 cm × 14 cm area around the wire mesh enclosure, and time spent in the corner along the wall opposite to the social interaction zone were measured. From these two stages, an interaction ratio was calculated (SI = time spent interaction zone, target present/time spent interaction zone, and target absent). Animals were defined as susceptible if SI < 1, and resilient if SI > 1. In a subset of studies, for acute stimulation, experimental mice underwent optogenetic stimulation to activate or inhibit DR serotonergic neurons, during both the target absent and target present sessions of the social interaction test. For chronic stimulation, mice received blue light stimulation two times a day (08:00 and 20:00, 10 min per session) in their home cage for 1 week.

### Open field

Mice were placed into the central zone of a white Plexiglas open field arena (40 × 40 × 30 cm) and were permitted to freely explore for 30 min. For optogenetic studies, the 30 min session was divided into three continuous 10-min epochs consisting of stimulation off, stimulation on, and stimulation off periods^56^. The following parameters were automatically calculated using DigBehv-002 animal behavior tracking software: the time spent in the central zone and the distance traveled.

### Elevated plus maze

To investigate the effects of optical stimulation on anxiety-like behavior, mice were placed in the center of a plastic elevated plus maze consisting of two open arms (10 × 50 cm) and two closed arms (10 × 50 cm) extending from a center platform (10 × 10 cm). The maze was elevated 50 cm off the ground. Animals were allowed to freely explore the maze during a 15 min session, divided into three continuous 5 min blocks, with light delivered only during the second block. The time spent in the open arms was automatically quantified by the same video tracking software (Ethovision XT 10, Noldus).

### Tail suspension test

Mice were suspended by the tail using adhesive tape applied 1 cm from the tip of the tail from a horizontal bar (roughly 50 cm above ground). Plastic tubes were placed around the tails of mice to prevent such tail climbing behavior. Mice were considered immobile without initiated movements (immobility was considered to include passive swaying). For optogenetic experiments, mice were subjected to two separate 5 min bouts (light OFF, then light ON) separated by a 5-min of rest period^42^.

### Sucrose preference test

Mice were gently introduced into a chamber equipped with two contact lickometers connected to two bottles on opposite walls. During the 60-min test, light delivery between 30 and 60 min only occurred if sucrose preference >30% in the first 30-min baseline epoch. Sucrose preference scores were measured during each session. Otherwise, the test was repeated on a different day^49^. Animals were water-restricted overnight before the experiment.

### Statistics and reproducibility

No specific method was used to predetermine the ideal sample size, or to randomly assign subjects into experimental groups^43,57–59^. All experiments were repeated independently with similar results at least three times. For comparisons with only two groups, we used a paired *t*-test or one-way ANOVA (when there were only two means to compare, the *t*-test and the *F*-test are equivalent; the relation between ANOVA and *t* is given by *F* = *t*^2^), as indicated in the figure legends. Comparisons across more than two groups were performed using one-way ANOVA, and a two-way ANOVA was used when there was more than one independent variable. These multiple group comparisons were followed by a Fisher LSD post hoc test for two by two comparisons when appropriate. All statistical tests were two-sided. The data are presented as the means ± s.e.m. The standard error of the mean is indicated by error bars for each group of data. All statistical data and corresponding sample sizes are provided in the corresponding figure legends. All data were analyzed with SPSS software and GraphPad Prism 6 (GraphPad Software, Inc., USA). Statistical significance was set at *P < 0.05, **P < 0.01, ***P < 0.001.

### Reporting summary

Further information on research design is available in the Nature Research Reporting Summary linked to this article.

## Supplementary information

Supplementary Information

Reporting Summary

## Data Availability

The data that support the findings of this study are available from the corresponding author upon reasonable request. The source data underlying Figs. 2c, e, 3c, f, i, 4c–e, h, i, 5b, f, g, and 6b–d, f–h, and Supplementary Figs. 1b, f, i, 2c, e, f, 3d–k, 4b, d, 5a–d, 6a–d, 7a–d, 8a–f, 9c, g, and 11a–e are provided as a Source Data file.

## Source data

Source Data
