# Supplementary material for: A discrete serotonergic circuit regulates vulnerability to social stress
Source: Nat Commun. 2020 Aug 24;11:4218. doi: 10.1038/s41467-020-18010-w (PMC7445164; doi:10.1038/s41467-020-18010-w)
Supplement: Supplementary file 1 — Supplementary Information [file 41467_2020_18010_MOESM1_ESM.pdf]

A discrete serotonergic circuit regulates vulnerability to social stress

Zou et al.

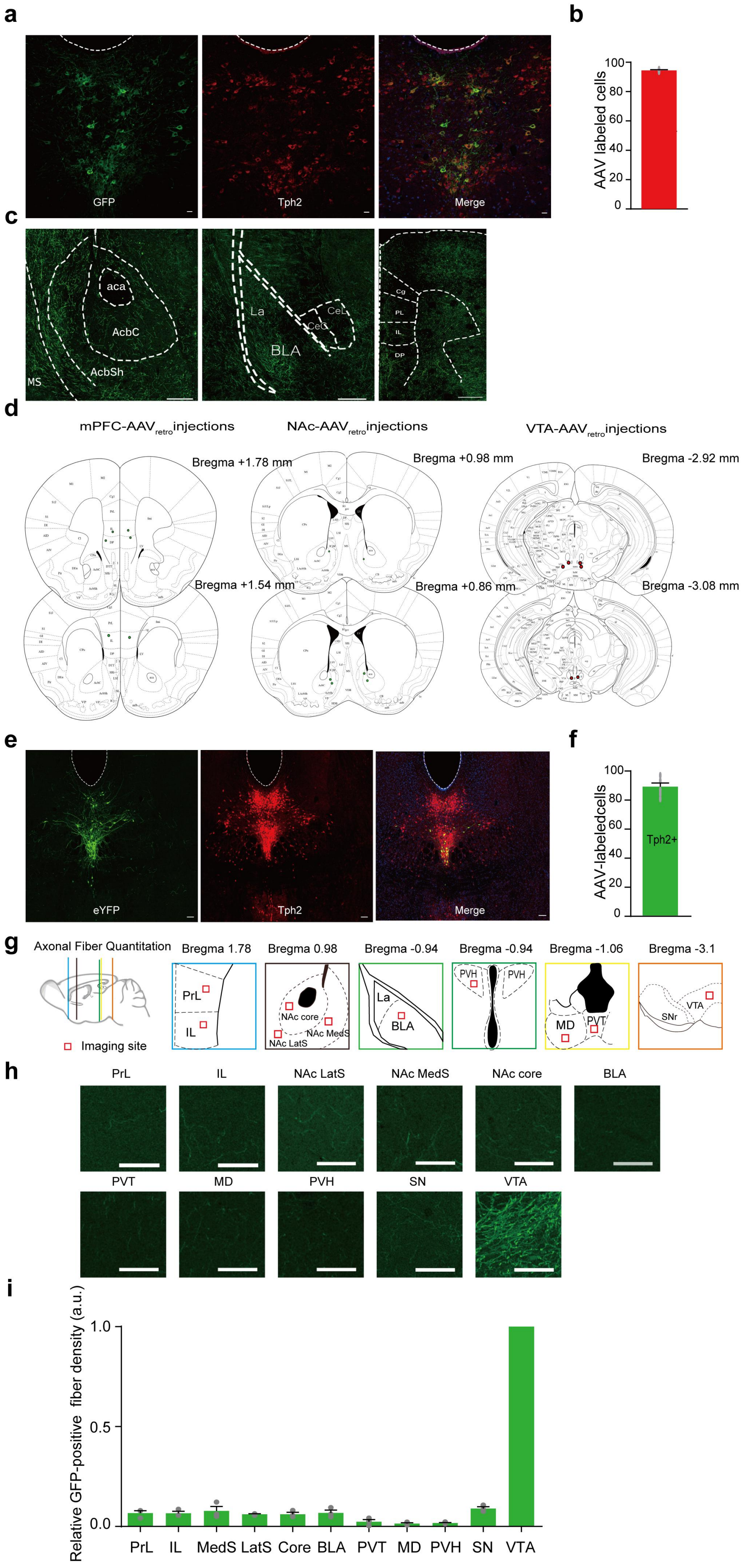

a
GFP
Tph2
Merge
b
AAV labeled cells
c
aca
AcbC
AcbSh
MS
La
CeL
CeC
BLA
Cg
PL
IL
DP
d
mPFC-AAV retro injections
NAc-AAV retro injections
VTA-AAV retro injections
Bregma +1.78 mm
Bregma +1.54 mm
Bregma +0.98 mm
Bregma +0.86 mm
Bregma -2.92 mm
Bregma -3.08 mm
e
eYFP
Tph2
Merge
f
AAV-labeledcells
Tph2+
g
Axonal Fiber Quantitation
Imaging site
Bregma 1.78
Bregma 0.98
Bregma -0.94
Bregma -0.94
Bregma -1.06
Bregma -3.1
PrL
IL
NAc core
NAc MedS
NAc LatS
La
BLA
PVH
PVH
MD
PVT
VTA
SNr
h
PrL
IL
NAc LatS
NAc MedS
NAc core
BLA
PVT
MD
PVH
SN
VTA
i
Relative GFP-positive fiber density (a.u.)
1.0
0.5
0.0
PrL
IL
MedS
LatS
Core
BLA
PVT
MD
PVH
SN
VTA

Supplementary Figure 1 | 5-HT$^{DR \rightarrow VTA}$ neurons represent an independent subpopulation. (a) Image showing the co-expression of AAV-DIO-eArch-GFP (green) in DR Tph2-positive serotonergic cells from Sert–Cre mice. Green, GFP expression; Red, anti-Tph2 staining. Scale, 20 µm. **(b)** Quantification shows that approximately 97.5% of AAV-DIO-eArch-GFP cells are Tph2+ (3 sections from two mice). **(c)** Other outputs of DR serotonergic neurons. Scale, 100 µm. **(d)** Reconstructions of virus injection locations in mice injected with AAV in the mPFC (left; green), the NAc (center; green) and the VTA (right; red). **(e)** Images of coronal sections containing the DR showing VTA-projecting serotonergic starter cells. Green, eYFP expression; Red, anti-Tph2 staining. Scale, 100 µm. **(f)** Quantification shows that approximately 89.4% of VTA-projecting serotonergic starter cells are Tph2+ (6 sections from two mice). **(g)** A sagittal view of several brain regions along the antero-posterior axis is taken for fiber quantitation analysis. Red boxes represent approximate locations of fluorescence quantification. **(h)** Representative images of fibers in target areas with GFP immunostaining after AAV_{retro}-DIO-Flp injection into the VTA and AAV-fDIO-eYFP into the DR. Scale: 100 µm. **(i)** Fiber quantitation of 5-HT$^{DR \rightarrow VTA}$ neurons. For analysis, 3 images were taken for each brain area within an individual animal (n = 3 animals) and averaged. Abbreviations: PrL, prelimbic cortex; IL, infralimbic cortex; DP, dorsal peduncular cortex; Cg, cingulate cortex; NAc, nucleus accumbens (MedS, medial shell; LatS, laterral shell; Core); BLA, basolateral amygdala; CeC, central amygdaloid nucleus, capsular part; Cel, central amygdaloid nucleus, lateral division; PVT, paraventricular thalamic nucleus; MD, mediodorsal nucleus of the thalamus; PVH, paraventricular hypothalamus; SN, substantia nigra; VTA, ventral tegmental area. The data are presented as the mean±s.e.m. Source data are provided as a Source Data file.

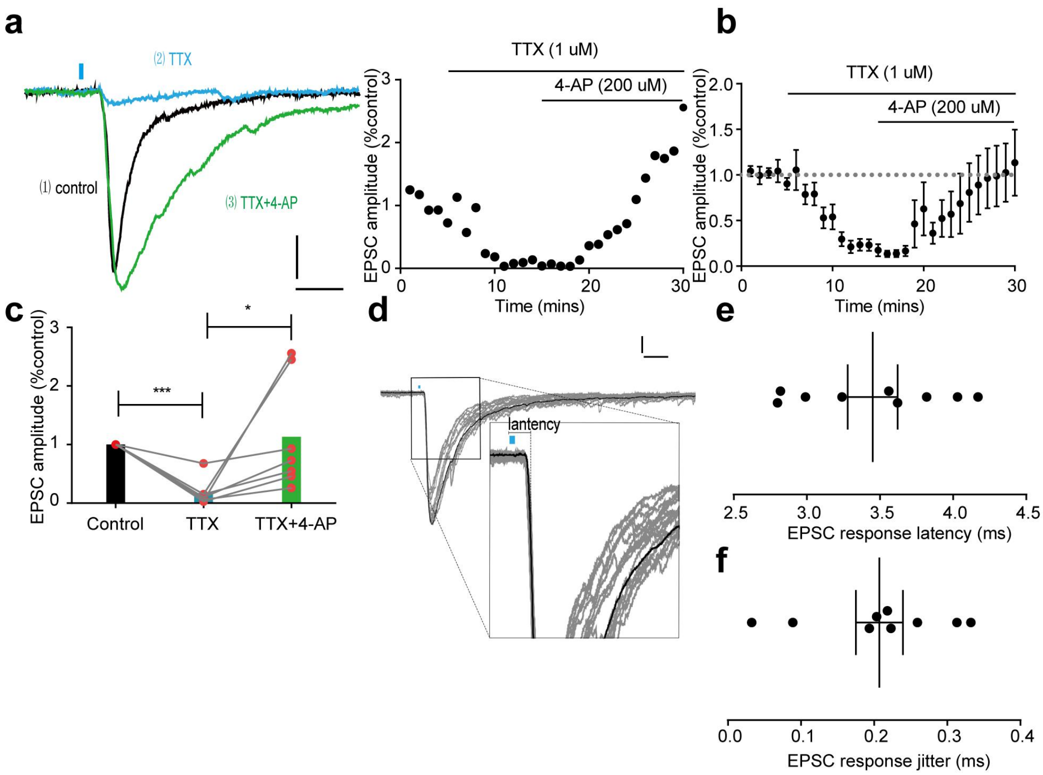

Supplementary Figure 2 | Monosynaptic transmission in 5-HT$^{DR\rightarrow VTA}$ pathway. (a) Left, a representative VTA neuron showing 5 ms photostimulation-evoked fast EPSCs (black trace). Optical-evoked EPSCs were eliminated by the bath application of 1 µM TTX (blue trace) and restored by the application of 200 µM 4-AP (green trace). Scale, 50 pA, 10 ms. Right, change in EPSC amplitude over time. Trace samples from the same neuron, with 60 s inter-trial intervals. Each dot presents the average of EPSCs over 1 min. (**b)** Summarized time course of the effects of TTX and 4-AP on the optical-evoked EPSCs (n = 7 cells/3 mice). **(c)** Summarized bar graph showing that the addition of 4-AP almost fully restored TTX inhibition. (n = 7 cells from 3 mice, one-way repeated measures ANOVA, *post hoc LSD,* $F_{1,6}$ = 39.508. control vs TTX: P = 0.000075, TTX vs TTX+4-AP: P = 0.044). **(d)** Example of a DR-driven excitatory response in a VTA neuron. Scale, 50 pA, 20 ms. The 5-fold enlarged inset shows the time-locked onset of individual responses (gray) to the light stimulation (blue) without any response failures. Averaged response shown in black. **(e, f)** Analysis of optical-evoked EPSCs show response latency (n = 9 cells from 3 mice) **(e)** and very low response jitter (standard deviation of latency, n = 9 cells from 3 mice) **(f),** both typical parameters for monosynaptic responses. Data are represented as mean ± SEM. * P < 0.05, ***P < 0.001. Source data are provided as a Source Data file.

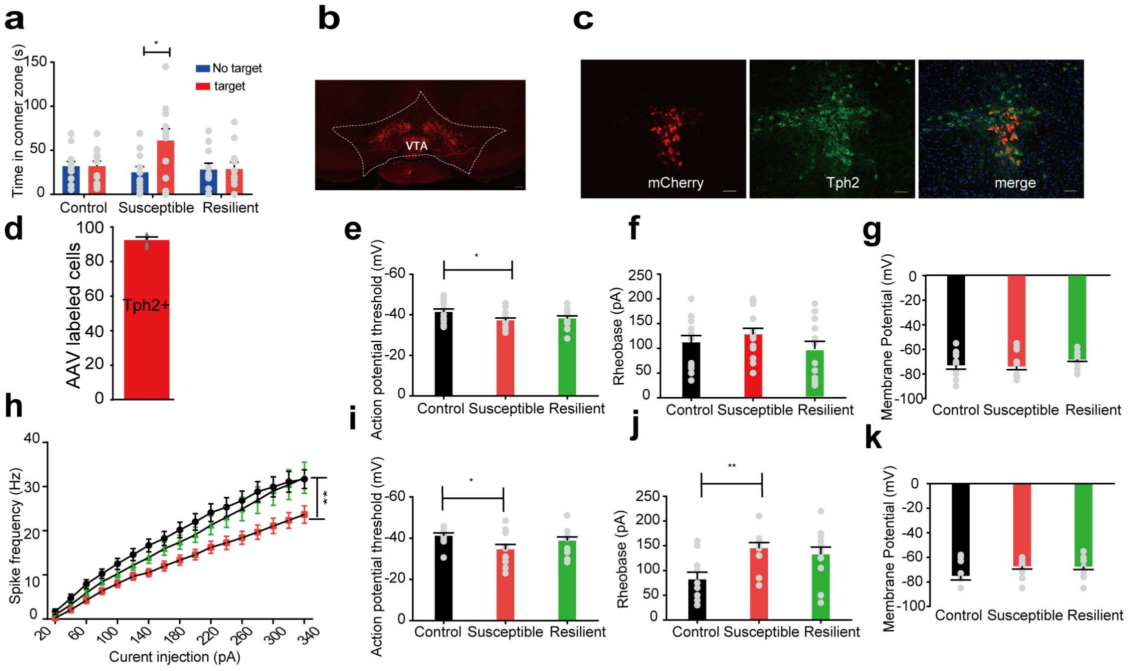

Supplementary Figure 3 | CSDS-induced changes in action potential and membrane properties in $5\text{-HT}^{DR\rightarrow VTA}$ neurons. **(a)** Time spent in the corner zone during the second phase in the presence of CD1 mice (target) was significantly increased compared to time spent the first phase with no target in susceptible mice (n = 11, one-way ANOVA, $F_{1,20}$ = 6.013, P = 0.024). **(b)** Confocal image of coronal section of a Sert-Cre mouse injected in VTA with AAV_{retro}-DIO-mCherry. Scale, 100 µm **(c)** Representative coronal confocal sections of retrogradely labeled (DR-VTA) cell bodies in DR of a Sert-Cre mouse; red, retrogradely labeled DR-VTA neurons; green, anti-Tph2 staining. Scale, 50 µm. **(d)** Quantification shows that approximately 96.4% of VTA-projecting starter cells are $Tph2^{+}$ (4 sections from two mice). **(e-g)** Comparisons of action potential thresholds (**e**, n = 14, 14, and 14 cells from 5, 4, and 4 control, resilient, and susceptible animals, respectively; one-way ANOVA, *post hoc LSD*, $F_{2,39}$ = 2.843, control vs susceptible: P = 0.029), rheobase (minimum current required to elicit action potential) **(f)** and resting membrane potential **(g)** for $5\text{-HT}^{DR\rightarrow VTA}$ neurons. **(h)** Input-output curve showing the spike frequency (Hz) of $5\text{-HT}^{DR\rightarrow VTA}$ neurons in response to a series of depolarizing current (pA) injections. Same as Fig **3i**, but with synaptic blockers CNQX, APV and bicuculline (n = 12, 12, and 12 cells from 4, 3, and 3 control, resilient, and susceptible animals, respectively; two-way ANOVA, *post hoc LSD*, $F_{2,33}$ = 4.782, control vs susceptible: P = 0.005). **(i-k)** Comparisons of action potential thresholds (**i**, one-way ANOVA, *post hoc LSD*, $F_{2,30}$ = 2.822, control vs susceptible: P = 0.027), rheobase (**j**, minimum current required to elicit action potential; one-way ANOVA, *post hoc LSD*, $F_{2,30}$ = 5.46, control vs susceptible: P = 0.004) and resting membrane potential **(k)** for $5\text{-HT}^{DR\rightarrow VTA}$ neurons. Data are represented as mean ± SEM. * P < 0.05, ** P < 0.01. Source data are provided as a Source Data file.

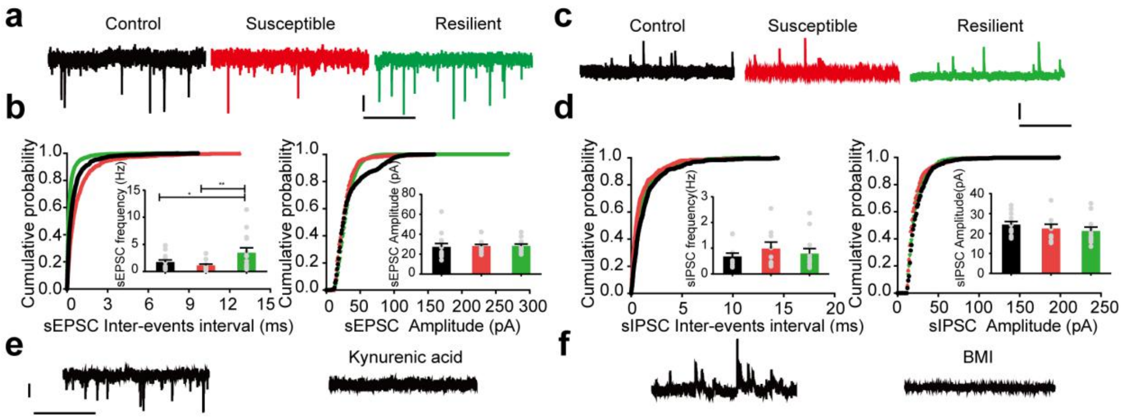

Supplementary Figure 4 | CSDS-induced changes in spontaneous synaptic activity in 5-HT$^{DR\rightarrow VTA}$ neurons. (a-d) Changes in spontaneous excitatory **(a, b)** and inhibitory **(c, d)** postsynaptic currents (sEPSC, sIPSC, respectively) in 5-HT$^{DR\rightarrow VTA}$ neurons from control (black), susceptible (red) and resilient (green) mice. Scale in **(a, c)**: 10 pA, 1 s. **(b, d)** Cumulative distribution of interevent intervals and average frequencies (left) and the cumulative distribution of amplitudes and average amplitudes (right). CSDS induced increases in sEPSC frequency (n = 14, 14, and 13 cells from 6, 6, and 5 control, resilient and susceptible animals, respectively; one-way ANOVA, *post hoc LSD*, $F_{2,38}$ = 4.289, control vs resilient: P = 0.043, susceptible vs resilient: P = 0.008) but not amplitude in 5-HT$^{DR\rightarrow VTA}$ neurons from resilient animals. **(e, f)** The sEPSCs or sIPSCs were blocked by the generic glutamate receptor blocker kynurenic acid (3 mM) or the GABAA receptor antagonist bicuculine (20 µM), respectively. Scale in **(e)** **(f)**, 10 pA, 1 s. Data are represented as mean ± SEM. * P < 0.05; ** P < 0.01. Source data are provided as a Source Data file.

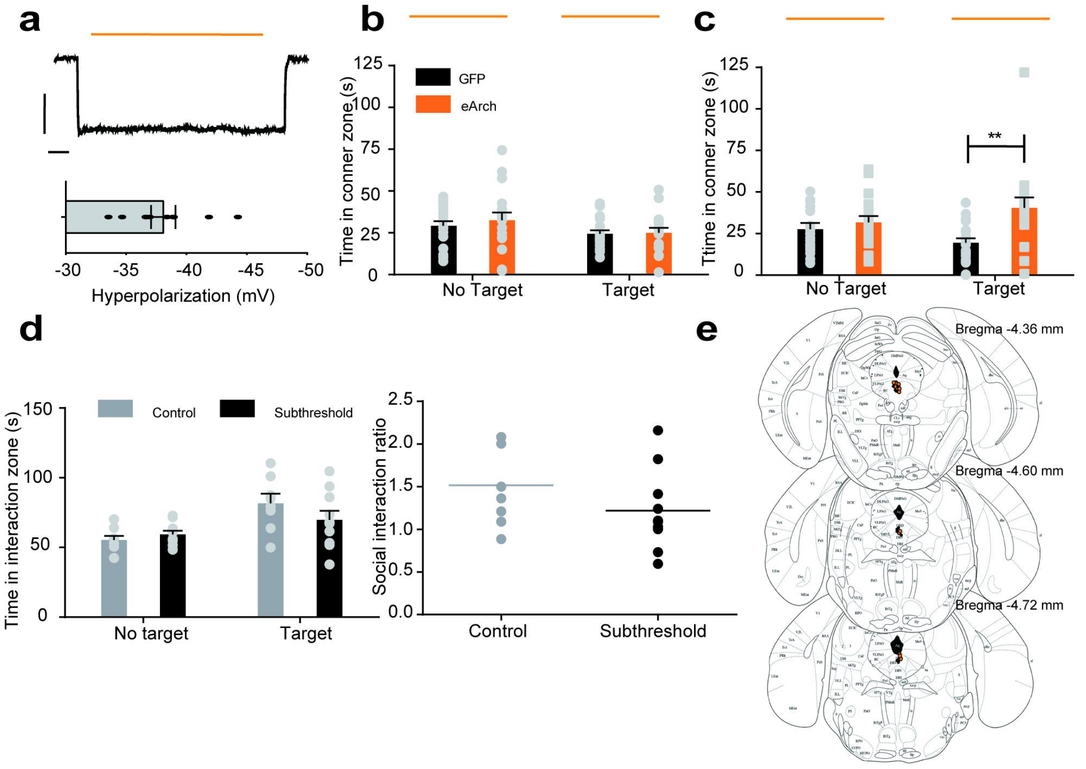

Supplementary Figure 5 | Optical inhibition of DR serotonergic neurons following subthreshold social defeat increased corner zone time. (a) Whole-cell current clamp recordings (top) of DR serotonergic neurons in response to 593 nm light (yellow lines) in DR slices showed that yellow light drove hyperpolarization. Summary plot (bottom) showing light-evoked robust hyperpolarization (*n* = 10 neurons from 2 mice). Scale, 20 mV, 1 s. **(b)** No significant difference was observed in the corner zone time during the silencing of DR serotonergic neurons in stress-naïve mice (eArch, *n* = 17 mice, GFP, *n* = 19 mice; one-way ANOVA, $F_{1,34} = 0.082$, $P = 0.776$). **(c)** Inhibition of DR serotonergic neurons following subthreshold defeat stress exposure during a 2nd social interaction test resulted in animals spending more time in the corner zone than control mice (eArch, *n* = 17 mice, GFP, *n* = 19 mice; one-way ANOVA, $F_{1,34} = 10.248$, $P = 0.003$). **(d)** Mice that underwent subthreshold social defeat spent roughly the same amount of time in the interaction zone, in the presence of a social target, as undefeated control mice (Control, *n* = 8 mice, Subthreshold, *n* = 10 mice; one-way ANOVA, interaction: $F_{1,16} = 1.58$, $P = 0.227$, SI ratio, $F_{1,16} = 1.796$, $P = 0.199$). **(e)** Histological verification of optical-fiber implant locations (eArch-mice: orange circles and GFP-mice: black circles) above the DR. Data are represented as mean ± SEM. $^{**}P < 0.01$. Source data are provided as a Source Data file.

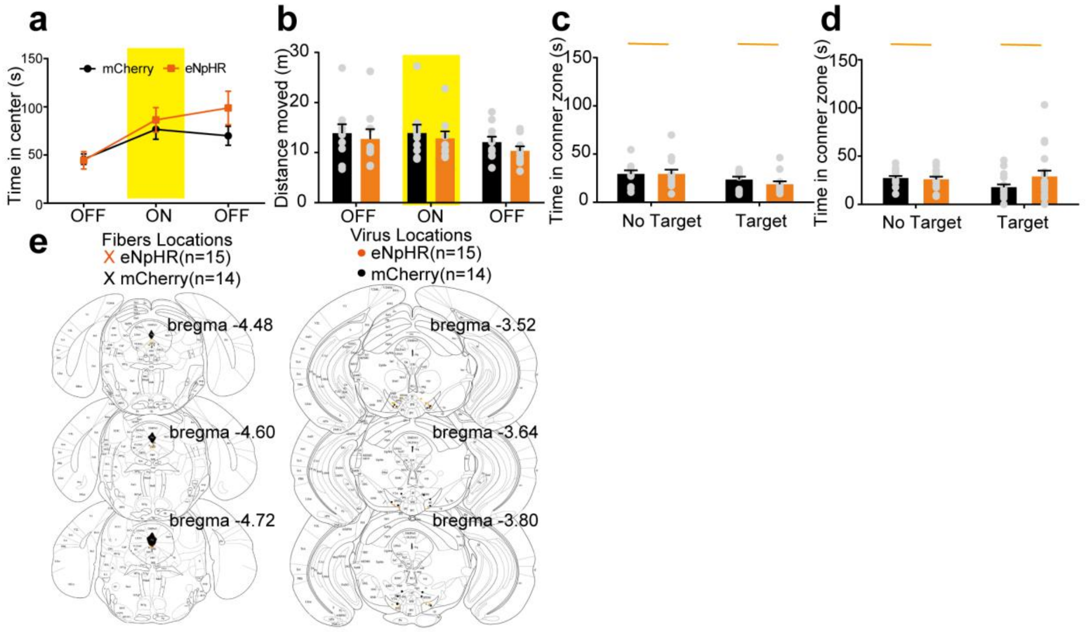

Supplementary Figure 6 | Acute inhibition of 5-HT$^{DR\rightarrow VTA}$ neurons had no effect on time spent in corners or on locomotion activity. (a, b) Inhibition of 5-HT$^{DR\rightarrow VTA}$ neurons had no effect on anxiety (eNpHR, n = 9 mice, mCherry, n = 10 mice; one-way ANOVA, $F_{1,17}$ = 0.371, P = 0.55) **(a)** or locomotor activity (eNpHR, n = 9 mice, mCherry, n = 10 mice; one-way ANOVA, $F_{1,17}$ = 0.23, P = 0.637) **(b)** in the open field test. **(c)** Inhibition of 5-HT$^{DR\rightarrow VTA}$ neurons had no effect on time spent in corners (eNpHR, n = 13 mice, mCherry, n = 11 mice; one-way ANOVA, $F_{1,22}$ = 1.269, P = 0.272). **(d)** Inhibition of 5-HT$^{DR\rightarrow VTA}$ neurons following subthreshold social defeat stress had no effect on the time spent in corners (eNpHR, n = 18 mice, mCherry, n = 17 mice; one-way ANOVA, $F_{1,33}$ = 2.562, P = 0.119). **(e)** Histological verification of bilateral virus injection locations (eNpHR-mice: orange circles and mCherry-mice: black circles) in the VTA and optic fiber implant locations (eNpHR-mice: orange crosses and mCherry-mice: black crosses) above the DR. Data are represented as mean ± SEM. Source data are provided as a Source Data file.

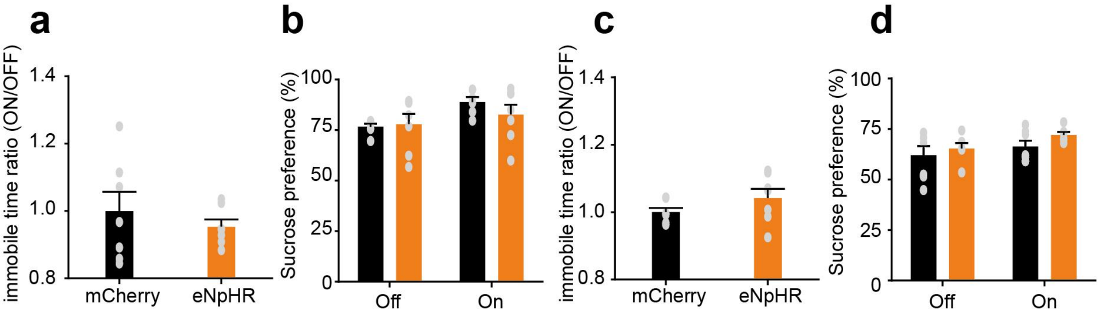

Supplementary Figure 7 | Acute inhibition of 5-HT$^{DR→VTA}$ neurons had no effect on TST or SPT. **(a, b)** No effect of acute inhibition of 5-HT$^{DR→VTA}$ neurons on TST (eNpHR, n = 7 mice, mCherry, n = 7 mice; one-way ANOVA, $F_{1,12}$ = 0.578, P = 0.462) **(a)**, or SPT (eNpHR, n = 7 mice, mCherry, n = 6 mice; one-way ANOVA, $F_{1,11}$ = 1.122, P = 0.312) **(b)** in stress-naïve mice. **(c, d)** No change in behavioral phenotypes in TST (eNpHR, n = 7 mice, mCherry, n = 7 mice; one-way ANOVA, $F_{1,12}$ = 1.978, P = 0.185) **(c)**, or SPT (eNpHR, n = 7 mice, mCherry, n = 6 mice; one-way ANOVA, $F_{1,11}$ = 2.706, P = 0.128) **(d)** following subthreshold defeat stress with acute inhibition of 5-HT$^{DR→VTA}$ neurons. Data are represented as mean ± SEM. Source data are provided as a Source Data file.

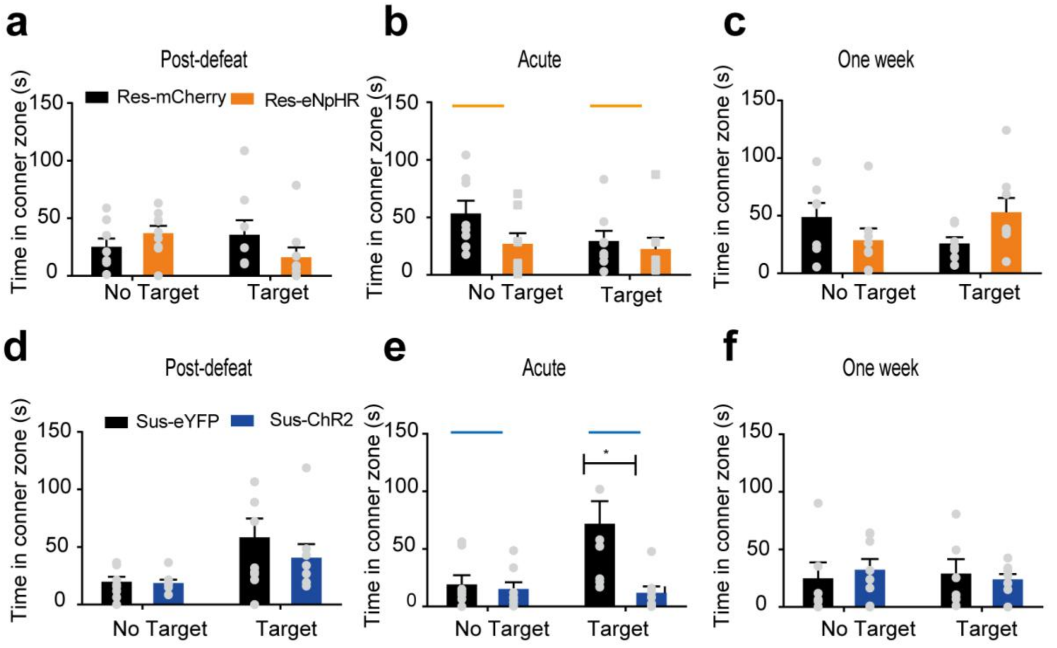

Supplementary Figure 8 | Detailed results of the social interaction test in resilient and susceptible mice with either the acute or chronic optogenetic stimulation of the 5-HT$^{DR\rightarrow VTA}$ circuit. (a, b, c) Time spent mice expressing either mCherry or eNpHR in the 5-HT$^{DR\rightarrow VTA}$ circuit in the corner zones after acute or 1 week stimulation (**a**, eNpHR, n = 9 mice, mCherry, n = 8 mice; one-way ANOVA, $F_{1,15}$ = 1.714, P = 0.21; **b**, eNpHR, n = 8 mice, mCherry, n = 8 mice; one-way ANOVA, $F_{1,14}$ = 0.272, P = 0.61; **c**, eNpHR, n = 8 mice, mCherry, n = 7 mice; one-way ANOVA, $F_{1,13}$ = 3.593, P = 0.08). **(d, e, f)** Time spent in corner zones in mice expressing either mCherry or ChR2 in a 5-HT$^{DR\rightarrow VTA}$ circuit after acute or 1 week stimulation. (**d**, eYFP, n = 9 mice; ChR2, n = 8 mice; one-way ANOVA, $F_{1,15}$ = 0.74, P = 0.403; **e**, eYFP, n = 8 mice; ChR2, n = 8 mice; one-way ANOVA, target: $F_{1,14}$ = 8.620, P = 0.011; **f**, eYFP, n = 6 mice; ChR2, n = 8 mice; one-way ANOVA, $F_{1,12}$ = 0.175, P = 0.683). Data are represented as mean ± SEM. *P < 0.05. Source data are provided as a Source Data file.

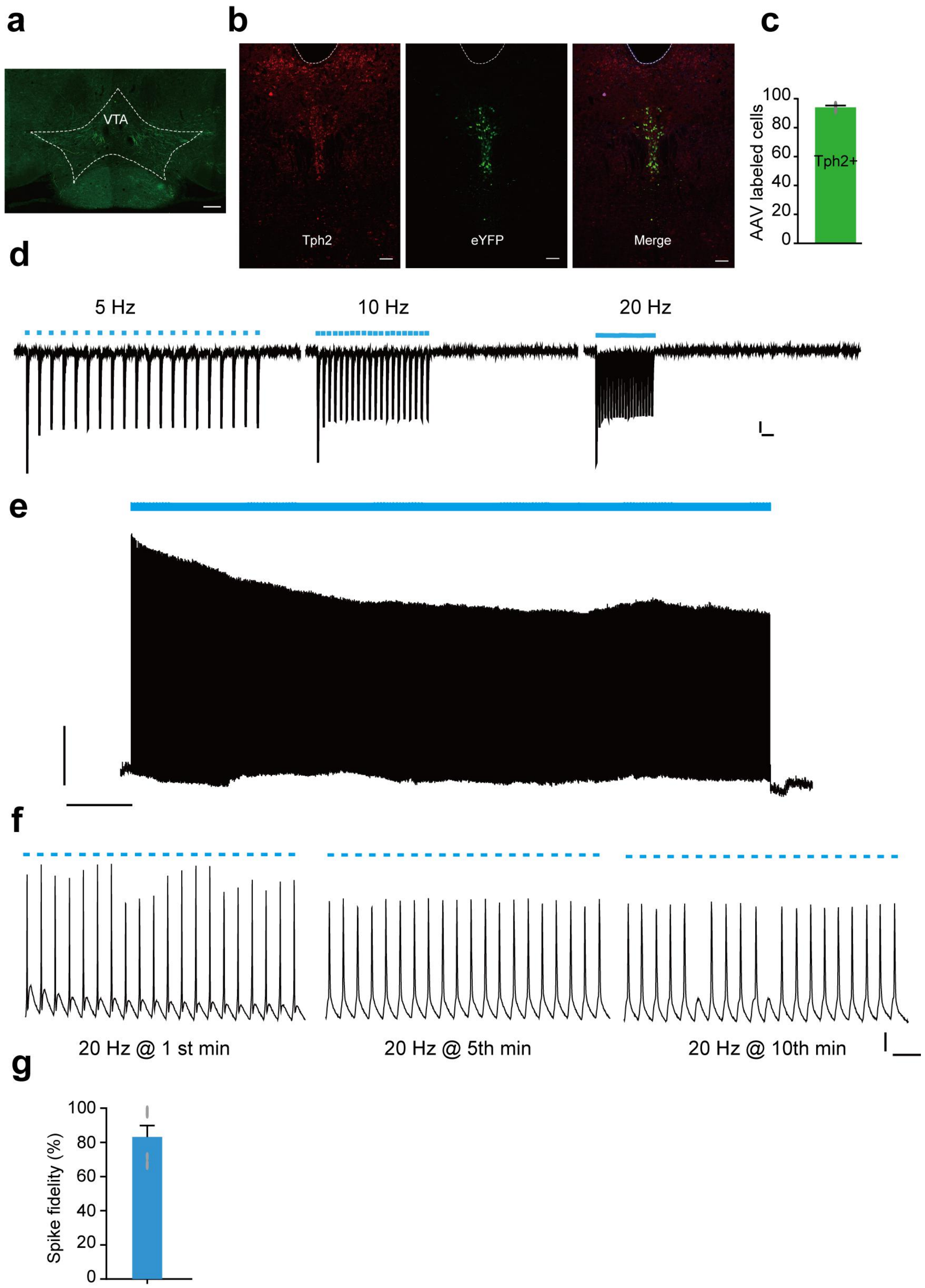

Supplementary Figure 9 | 5-HT$^{DR \rightarrow VTA}$ neurons did not enter depolarization block with 20 Hz optical stimulation. (a) Confocal image of coronal section of a Sert-Cre mouse injected in VTA with AAV$_{retro}$-DIO-ChR2-eYFP. Scale, 100 µm. **(b)** Representative coronal confocal sections of retrogradely labeled (DR-VTA) cell bodies in the DR of a Sert-Cre mouse. Green, retrogradely labeled DR-VTA neurons; Red, anti-Tph2 staining. Scale, 100 µm. **(c)** Quantification shows that approximately 98% of AAV-DIO-eArch-GFP cells are Tph2^{+} (3 sections from two mice). **(d)** Trains of brief light pulses (blue dots) produced temporally precise inward

photocurrents at frequencies of 5, 10 and 20 Hz. Scale, 100 pA, 200 ms. **(e)** 20 Hz 5-msec pulses of blue light evoked firing of action potentials with high fidelity throughout the 10-minute stimulation period. Scale, 20 mV, 1 min. **(f)** Representative traces from a 5-HT$^{DR \rightarrow VTA}$ neuron responding to 20 Hz blue light illumination given for 10 min. Scale, 10 mV, 100 ms. **(g)** Quantification of stimulation-induced action potentials over ten minutes of optical stimulation (n = 6 cells/4 mice). Data are represented as mean ± SEM. Source data are provided as a Source Data file.

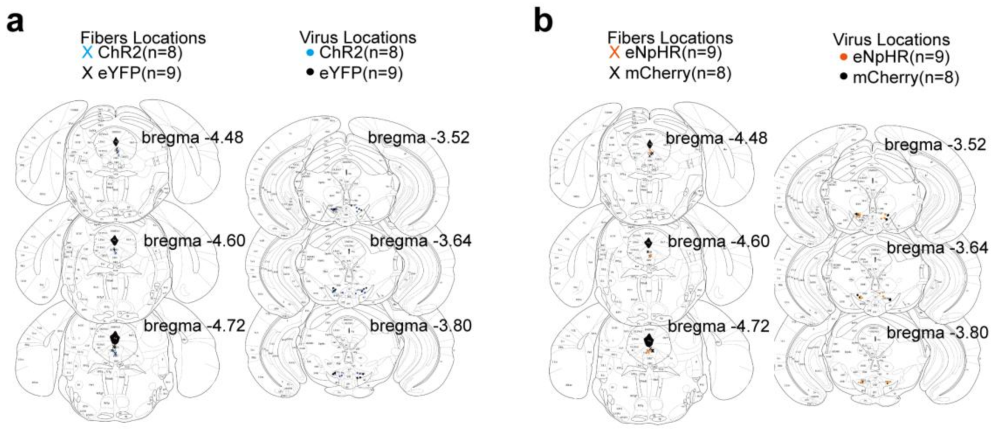

Supplementary Figure 10 | Histological verification of fiber tip and virus injection locations. (a-b) Histological verification of bilateral virus injection locations (ChR2-mice: blue circles, eNpHR-mice: orange circles, eYFP-mice and mCherry-mice: black circles) in the VTA and optic fiber implant locations (ChR2-mice: blue crosses, eNpHR-mice: orange crosses, eYFP-mice and mCherry-mice: black crosses) above the DR.

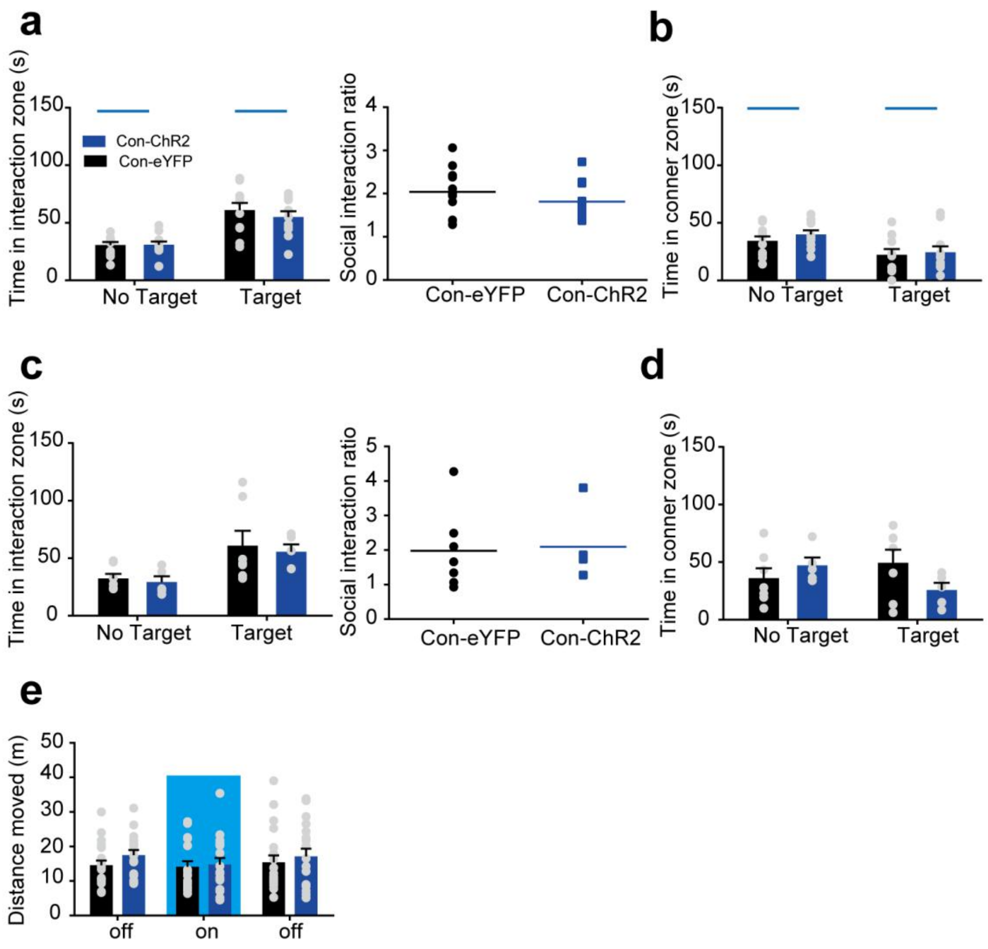

Supplementary Figure 11 | Detailed results of the social interaction test in control mice with either acute or chronic optogenetic stimulation of the 5-HT$^{DR\rightarrow VTA}$ circuit. (a-d) In control mice, either the acute **(a,b)** or chronic **(c,d)** stimulation of the 5-HT$^{DR\rightarrow VTA}$ circuit had no effect on the time spent in the interaction zone with the target **(a, c)** or the time spent in the corners **(b, d)** (**a,** eYFP, n = 10 mice, ChR2, n = 11 mice; one-way ANOVA*,* interaction: $F_{1,19}$ = 1.334, P = 0.262, SI ratio, $F_{1,19}$ = 0.694, P = 0.415; **b**, eYFP, n = 10 mice, ChR2, n = 11 mice; one-way ANOVA*,* $F_{1,19}$ = 0.481, P = 0.496; **c**, eYFP, n = 7 mice, ChR2, n = 5 mice; one-way ANOVA*,* interaction: $F_{1,10}$ = 0.109, P = 0.748, SI ratio, $F_{1,10}$ = 0.052, P = 0.824; **d**, eYFP, n = 7 mice, ChR2, n = 5 mice; one-way ANOVA*,* $F_{1,10}$ = 2.636, P = 0.136). **(e)** Photostimulation of the 5-HT$^{DR\rightarrow VTA}$ circuit had no effect on locomotor activity in the open field (eYFP, n = 20 mice, ChR2, n = 18 mice; one-way ANOVA*,* $F_{1,36}$ = 0.071, P = 0.791). Data are represented as mean ± SEM.Source data are provided as a Source Data file.
